# Studies on sugar transporter CRT1 reveal new characteristics that are critical for cellulase induction in* Trichoderma reesei*

**DOI:** 10.1186/s13068-020-01797-7

**Published:** 2020-09-14

**Authors:** Sami Havukainen, Mari Valkonen, Kari Koivuranta, Christopher P. Landowski

**Affiliations:** VTT Technical Research Center of Finland Ltd, Tietotie 2, 02150 Espoo, Finland

**Keywords:** *Trichoderma reesei*, Transmembrane transport, Cellobiose transporter, Cellulase induction

## Abstract

**Background:**

*Trichoderma reesei* is an ascomycete fungus that has a tremendous capability of secreting extracellular proteins, mostly lignocellulose-degrading enzymes. Although many aspects of the biology of this organism have been unfolded, the roles of the many sugar transporters coded in its genome are still a mystery with a few exceptions. One of the most interesting sugar transporters that has thus far been discovered is the cellulose response transporter 1 (CRT1), which has been suggested to be either a sugar transporter or a sensor due to its seemingly important role in cellulase induction.

**Results:**

Here we show that CRT1 is a high-affinity cellobiose transporter, whose function can be complemented by the expression of other known cellobiose transporters. Expression of two sequence variants of the *crt1* gene in *Saccharomyces cerevisiae* revealed that only the variant listed in the RUT-C30 genome annotation has the capability to transport cellobiose and lactose. When expressed in the $\Delta$*crt1* strain, the variant listed in the QM6a genome annotation offers partial complementation of the cellulase induction, while the expression of the RUT-C30 variant or cellobiose transporters from two other fungal species fully restore the cellulase induction.

**Conclusions:**

These results add to our knowledge about the fungal metabolism of cellulose-derived oligosaccharides, which have the capability of inducing the cellulase production in many species. They also help us to deepen our understanding of the *T. reesei* lactose metabolism, which can have important consequences as this sugar is used as the inducer of protein secretion in many industrial processes which employ this species.

## Background

As the fossil fuel reserves deplenish, there is growing need for renewable fuels. Lignocellulose biomass is an abundant and renewable feedstock for second-generation biofuels. In order to utilize lignocellulose biomass as raw material for biofuel production, its cellulose and hemicellulose fractions have to be saccharified into fermentable sugars by cellulase and hemicellulase enzymes [1]. These sugars can then be utilized by yeast or another microorganism for the production of the biofuels, such as ethanol or butanol. Due to the recalcitrant nature of lignocellulose, an enzyme mixture with wide range of different kind of activities is required for this task [1]. Saprophytic organisms, which live by decaying lignocellulose biomass, produce a wide range of enzymes to support their lifestyle. Thus they are prime candidates for producing the enzyme mixtures needed for efficient degradation of lignocellulose biomass. Since enzymes account for a significant portion of the production costs of second-generation biofuels, ways of improving the production hosts are sought for [1].

*Trichoderma reesei* is an ascomycete fungus which has been known for its outstanding cellulase production capacity for over 60 years. Nowadays *T. reesei* is used for the industrial production of cellulase enzymes due to its high protein production capacity, with values up to 80 g/L reported in literature [2]. It has also been extensively studied for heterologous protein production for the same reason [3]. Although many aspects of the lignocellulose degradation mechanism of *T. reesei* have been studied for decades, there are still many open questions remaining to be answered.

One intriguing question about the physiology of filamentous fungi is the role of the many sugar transport proteins in these organisms. It has been predicted that two well-known cellulase producers, *Aspergillus niger* and *T. reesei*, harbor 256 and 113 sugar transporter proteins in their in silico proteome, respectively, in contrast to just 43 in yeast *Saccharomyces cerevisiae* [4]. In addition, in fungi the sugar transporter genes seem to be more divergent than in yeast [5]. This is to be expected since the sugar substrate range of filamentous fungi is much broader than that of *S. cerevisiae*, which is able to grow on only few mono- and disaccharides. The saprotrophic lifestyle of filamentous fungi such as *T. reesei* requires the possibility to utilize many kinds of pentose and hexose sugars as well as oligosaccharides, that derive from lignocellulose breakdown and are present in only small concentrations in the natural habitat of the fungus [6].

It is thought that although cellulose as a solid substrate can cause induction of cellulase and hemicellulase production, the actual inducer is a soluble product cleaved from cellulose via basal cellulase activity, which is maintained at low levels even in non-inducing conditions [7]. Soluble cellulase inducers for *T. reesei* include cellobiose, lactose, sorbose and sophorose, whereas glucose represses the induction [7]. The mechanism of how the fungus senses the presence of the inducing sugar is not known yet, but the research hints towards the involvement of membrane proteins in this process. For example, in yeast *Saccharomyces cerevisiae*, glucose is sensed by receptor *GPR1* and two non-functional glucose transporters *SNF3* and *RGT2* [8]. In filamentous fungi, such as *Neurospora crassa* or *Aspergillus nidulans*, membrane-bound sugar transporter proteins have been shown to affect cellulase production [9, 10]. In *N. crassa*, deletion of genes coding for cellodextrin transporters CDT-1 or CDT-2 caused a marked decrease in the expression of cellulase genes *cbh-1* and *gh5-1* when either cellulose or cellobiose was used as inducer [9]. This indicates that these transporters have a role in the cellulase induction, either by transporting the inducer into the cell where it is sensed by some other enzyme or by sensing it itself [9]. Similar results were obtained from *A. nidulans*, where double deletion of cellobiose transporter genes *cltA* and *cltB* caused a markedly decreased cellulase activity and biomass formation when the fungus was grown on Avicel cellulose [10]. Additionally Znameroski et al. [9] showed that the expression of mutated forms of cellodextrin transporters which had markedly decreased cellobiose uptake activity rescued the cellulase induction in *N. crassa*. This could indicate that these transporters have a function in cellulose sensing independent of their transport activity (i.e., they would function as tranceptors).

Although fungal sugar transporters seem to have an important role in cellulase production, only few of them have been characterized in the literature. The previously mentioned cellodextrin transporters CDT-1 and CDT-2 were the first cellobiose transporters discovered from fungi. They were discovered by Galazka et al. in 2010 [8], who co-expressed them in yeast with intracellular $\upbeta$-glucosidase GH1-1 from *N. crassa*, allowing yeast to grow on cellobiose. Both of them could also support yeast growth on cellotriose and CDT-1 also on cellotetraose [8]. Kim et al. [11] further analyzed these transporters and discovered that CDT-1 is a proton symporter and CDT-2 a passive facilitator, although they both have very high affinities for cellobiose ($K_{\mathrm{m}}$ values 4 and 3.2 $\upmu$M, respectively) [8]. The third fungal $\upbeta$-linked disaccharide transporter was identified in 2012 by Fekete et al. [12] who discovered a lactose transporter lacpA from *A. nidulans*. This was followed by reports about the glucose/cellobiose transporter STP1, putative lactose transporter/cellulose sensor CRT1 and two other putative lactose transporters from *T. reesei* and cellodextrin transporters from *Penicillium oxalicum* [13–16]. Later another lactose transporter lacpB/cltB was discovered from *A. nidulans*, and this transporter as well as another transporter cltA were characterized as cellobiose transporters [10, 17].

Strategies for improving cellulase production by modification of sugar transport have been shown in the literature. For example, Wang et al. [18] proved that simple deletion of two high-affinity glucose transporters in *N. crassa* caused threefold increase in protein production when grown on Avicel cellulose. The upregulation of cellulase-encoding genes and of cellulase regulator *clr-2* was observed, while the expression of carbon catabolite repression regulator *cre-1* was downregulated. Similarly, deletion of glucose/cellobiose transporter STP1 improved cellulase production in *T. reesei* when grown on Avicel, possibly due to decreased glucose uptake [13]. Besides inhibiting glucose uptake by transporter deletions as done in these studies, other possible strategies for improving cellulase production would be to increase the uptake of the inducing sugar, such as lactose.

Of the *T. reesei* transporters, the previously mentioned putative lactose transporter/cellulose sensor CRT1 was shown to be important for cellulase induction, and thus manipulation of its expression could provide means for improving cellulase production [13, 14]. Sole deletion of *crt1* was enough to abolish cellulase production in *T. reesei*, whereas the same effect could be achieved by the deletion of two cellobiose transporters in both *N. crassa* and *A. nidulans* [9, 10, 13, 14]. Constitutive overexpression of CRT1 resulted in 1.5-fold higher *cbh1* and *eg1* expression on Avicel and earlier appearance of *cel7a* and *cel6a* transcripts on lactose [13, 14]. However, the function of CRT1 is not clear based on the available literature. When Zhang et al. [13] heterologously expressed the CRT1 in yeast, the resulting strain could not grow glucose or cellobiose. Therefore they concluded that CRT1 is a sensor for cellulose. Porciuncula et al. [15] also concluded that although CRT1 has an important role in cellulase induction, they do not believe that it has a direct role in the uptake of lactose or other inducing sugars. On the other hand, Ivanova et al. [14] concluded that CRT1 is a lactose transporter since the deletion strain showed a growth and protein production defect in lactose.

To unravel its role in cellulase induction and sugar transport, we characterized CRT1 via functional studies in *S. cerevisiae*. We expressed two different sequence variants of *crt1* with the first based upon the annotation of the QM6a genome assembly and the second based upon the annotation of the RUT-C30 genome assembly [19, 20]. We also tested the effects of *crt1* deletion and overexpression in *T. reesei* in different strain backgrounds and investigated if the effects of its deletion can be complemented by the expression of heterologous transporter proteins. In this way, we could test whether CRT1 itself is critical for sensing or transporting inducing sugars.

## Results

### Alternative annotation revealed new functionalities of CRT1

**Fig. 1:**
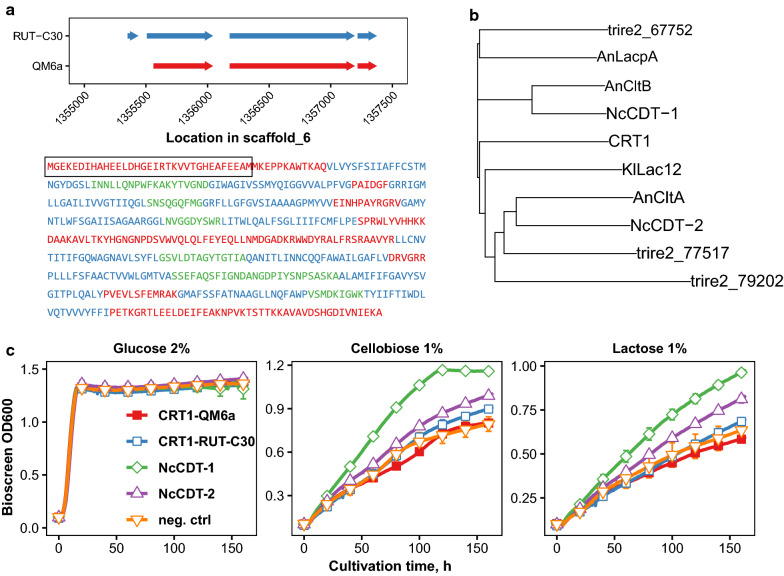
Alternative forms of CRT1 and their effect on yeast growth.** a** Comparison of genomic location of the exons of QM6a and RUT-C30 versions of *crt1* and the predicted localization of the protein (red = intracellular, blue = transmembrane, green = extracellular). Box presents the area which is missing from the QM6a annotation.** b** Phylogenetic analysis of cellobiose and lactose transporters whose functionality has been demonstrated from *N. crassa*, *A. nidulans* and from milk yeast *K. lactis* with their *T. reesei* homologs.** c** Growth curves of yeast strains in liquid cultures with cellobiose, glucose or lactose as the carbon sources. Growth curves were measured with a Bioscreen C incubator. Error bars present standard deviation between four biological replicates, and for the sake of clarity they and points are drawn for only every 20 h

We were intrigued by the conflicting findings in the literature about the transport activity of CRT1 and by its suggested role in cellulase induction, so we decided to heterologously express it in yeast to check for growth on different carbon sources. Two different versions of the gene were expressed, one based on the annotation of the QM6a genome assembly in the Joint Genome Institute (JGI) database (Trire2_3405, hereon referred to as CRT1-QM6a) and the other based on the annotation of the RUT-C30 genome assembly (TrireRUTC30_1:109243, hereon referred to as CRT1-RUT-C30) in the JGI database [19, 20]. The RUT-C30 annotation contains an additional exon and intron at the N-terminus of the protein. The N-terminal exon is located at nucleotides 1355351–1355395 at scaffold 6 of QM6a genome annotation, and additionally the second exon is extended to start at position 1355507 instead of 1355561 (Fig. 1a). This extra section does not contain any transmembrane segments and is predicted by TMHMM server to be located inside the cell similarly to the N-terminus of the QM6a version of the gene [21]. As reported before, *crt1* is in the same genomic region with transcription factor *clr2* (trire2_26163), which is involved in regulation of xylanase and pectinase genes, and these genes appear to be co-expressed based on transcriptome analysis done in the RUT-C30 strain [22, 23]. Phylogenetic alignment of the amino acid sequence of CRT1 with some of the well characterized fungal disaccharide transporters showed that it is quite distant from the *N. crassa* cellodextrin transporters CDT-1 and CDT-2, which have other homologs in *T. reesei* (Fig. 1b, alignment was done with MUSCLE [24]). Homologs of the passive facilitator CDT-2 include putative lactose transporters trire2_77517 and trire2_79202 [15], while closest functionally characterized homolog for the proton symporter CDT-1 is trire2_67752 [25].

Both versions of *crt1* were ordered as codon-optimized synthetic genes and expressed in yeast. As *S. cerevisiae* cannot metabolize cellobiose or lactose, the CEN.PK2-1D-based host strain for the growth studies was modified to express the codon-optimized gene for *N. crassa* $\upbeta$-glucosidase GH1-1. The GH1-1 has been shown to hydrolyze lactose by acting as a $\upbeta$-galactosidase, and the expression of CDT-1 and GH1-1 allowed yeast growth on lactose [26]. $\upbeta$-glucosidase assay of cell extracts prepared from the strains with and without GH1-1 expression showed that $\upbeta$-glucosidase activity was increased in the latter strain (data not shown). Yeast strains expressing different versions of CRT1 or *N. crassa* cellodextrin transporters were grown in liquid media to compare their growth phenotypes (Fig. 1c). The transporters were expressed with *PGK1* promoter from a centromeric plasmid with *URA3* selection. The negative control strain harbored a similar plasmid which expressed the gene for *T. reesei* intracellular $\upbeta$-glucosidase CEL1A (Trire2_120749).

To study the growth of the strains expressing different versions of CRT1 or *N. crassa* cellodextrin transporters we measured growth curves of the strains with the Bioscreen incubator. In liquid media with 1% cellobiose as the carbon source, improved growth was seen for strains expressing CDT-1 or CDT-2 (Fig. 1c). The strain expressing CRT1-RUT-C30 grew also slightly better in cellobiose medium than the strain expressing CRT1-QM6a, the growth of which resembled that of the negative control strain. In lactose medium, improved growth was seen by strains expressing *N. crassa* transporters. We also tested growth in liquid media with 0.1% cellotriose or cellotetraose as carbon sources (data not shown), which both seemed to rescue growth of CDT-1 expressing strain, while CDT-2 expressing strain grew only in cellotriose medium as was also previously reported [8]. However, expression of either version of the *crt1* gene did not rescue growth on cellotriose or cellotetraose.

### Transport properties of CRT1

**Fig. 2:**
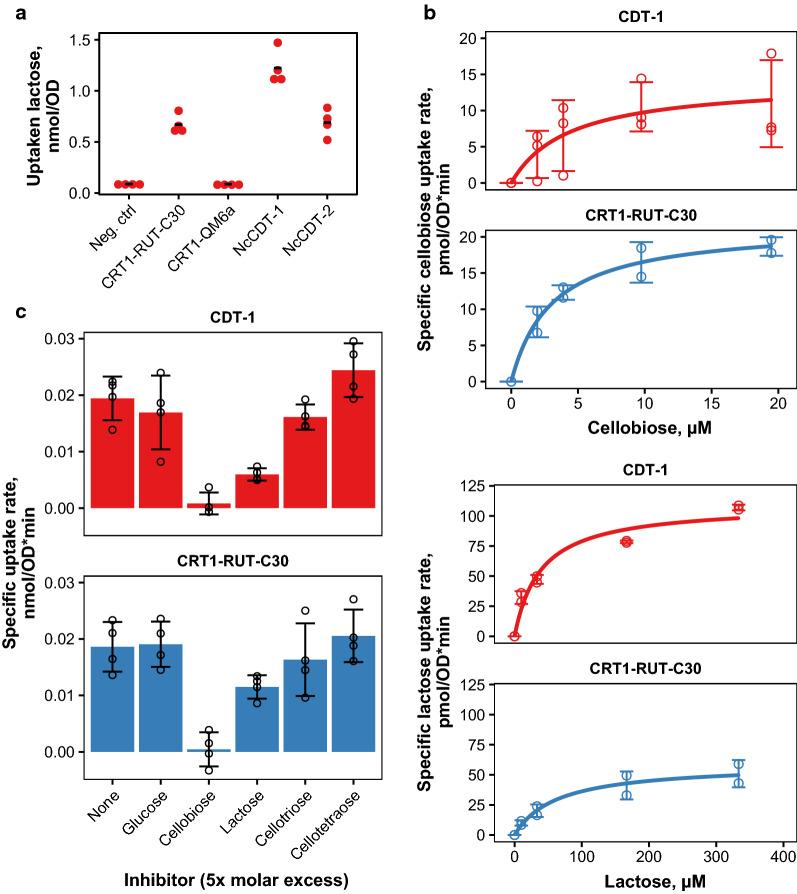
Transport studies with radiolabeled sugars.** a** Amount of lactose uptaken by yeast strains expressing *N. crassa* $\upbeta$-glucosidase and different transporters during the 20-min assay with 5 mM lactose. The dots present the values obtained for the biological replicates (*n* = 4) and the bars present their average.** b** Kinetics of cellobiose (top) or lactose (bottom) transport by yeast strains expressing CRT1-RUT-C30 or CDT-1. Error bars present standard deviation between 2–3 independent experiments.** c** Inhibition of cellobiose uptake by CDT-1 or CRT1 by different sugars in yeast. Cellobiose concentration in the reaction was 5 mM and inhibitor concentration was 25 mM. Error bars present standard deviation from 2 replicates from 2 individual experiments (*n* = 4)

After having evidence of growth and thus transport activity, we decided to measure sugar uptake activities of yeast strains expressing the two CRT1 versions and other known transporters CDT-1 and CDT-2. Based upon previous literature describing CRT1 as a lactose transporter, we first chose to investigate the uptake of lactose. We also investigated whether any of the transporters could transport glucose as well. As an initial experiment, we tested the amount of radioactivity obtained by the strains during the 20-min assay with 5 mM lactose. As shown in Fig. 2a, higher lactose uptake was seen for the strains which expressed CDT-1, CDT-2 or CRT1-RUT-C30 when compared to the negative control and to the strain expressing CRT1-QM6a (*t*-test: *p* < 0.001 for CDT-1 and CRT1-RUT-C30, *p* < 0.01 for CDT-2).

Since the used background strain does not have deletions of the endogenous hexose transporters, we transformed the plasmids into a hexose-negative yeast strain. This strain also expressed the GH1-1 $\upbeta$-glucosidase to allow growth on cellobiose and lactose. Growth tests in this background revealed that none of the transporters rescued growth on glucose, but CRT1-RUT-C30 and both of the *N. crassa* transporters could rescue growth on cellobiose or lactose (data not shown). Uptake tests performed in the hexose-negative background indicated that strains expressing CRT1-RUT-C30, CDT-1 or CDT-2 could uptake higher amount of glucose than the control strain or the strain expressing CRT1-QM6a, although the amount of uptaken sugar was lower for glucose than for lactose (data not shown).

Next we wanted to see how the transport kinetics of CRT1 would differ from those of CDT-1, which is probably the most characterized fungal cellodextrin transporter. Kinetics of both CDT-1 and CDT-2 have been characterized by Galazka et al. [8] who reported that both of these transporters have very high affinity for cellobiose with $K_{\mathrm{m}}$ values in the range of few $\upmu$M. Transport kinetics with either cellobiose or lactose in our study showed that both CRT1 and CDT-1 are indeed high-affinity transporters, with both having lower $K_{\mathrm{m}}$ for cellobiose than lactose. Therefore, CRT1 is also a cellobiose transporter rather than just a lactose transporter (Fig. 2b). On cellobiose, $V_{\mathrm{max}}$ and $K_{\mathrm{m}}$ values were 14 ± 3.7 pmol nmol$^{-1}$ OD$^{-1}$ and 4.4 ± 3.3 $\upmu$M for CDT-1, and 21.7 ± 1.5 pmol nmol$^{-1}$ OD$^{-1}$ and 3.1 ± 0.7 $\upmu$M for CRT1. However, on lactose, $V_{\mathrm{max}}$ and $K_{\mathrm{m}}$ values were 109.6 ± 8.4 pmol nmol$^{-1}$ OD$^{-1}$ and 38.8 ± 11.8 $\upmu$M for CDT-1, and 59.1 ± 7.6 pmol nmol$^{-1}$ OD$^{-1}$ and 62.9 ± 27.4 $\upmu$M for CRT1. The $K_{\mathrm{m}}$ value for CDT-1 is comparable to that published by Galazka et al. [8] ($K_{\mathrm{m}}$ 4 $\upmu$M). However, it must be kept in mind that the $V_{\mathrm{max}}$ values mentioned here are specific for this system since they depend on the expression level of the transporter and should not be compared to other values.

The substrate binding properties of CDT-1 and CRT1 were further tested with inhibition assays, where the uptake of labeled cellobiose was measured in the presence of non-radiolabeled sugars. The uptake by either CRT1 or CDT-1 was not inhibited by glucose, but it was inhibited by cellobiose and to some extent by lactose and cellotriose (Fig. 2c). The inhibition by lactose seemed to be stronger for CDT-1 (*t*-test: *p* < 0.01 for none vs lactose) than for CRT1 (*t*-test: *p* < 0.05 for none vs lactose). Also, as reported before [11], the plasma membrane uncoupler carbonyl cyanide 3-chlorophenylhydrazone (CCCP) inhibited transport by CDT-1 and also by CRT1, which would indicate that CRT1 functions as a proton symporter similarly to CDT-1 (data not shown). We did not pursue the kinetics and inhibition studies with CDT-2 since as a passive facilitator it is a different kind of transporter than CDT-1 and CRT1.

### Overexpression of *crt1* rescues protein secretion defect on lactose

**Fig. 3:**
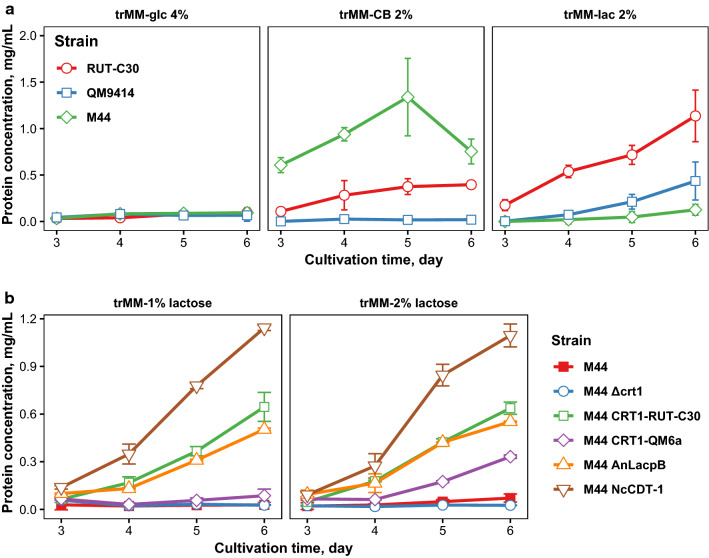
Protein production by parental strains and the effect of CRT1 overexpression.** a** Protein secreted into culture medium by different mutant strains growing on minimal medium with indicated carbon sources. Error bars present standard deviation between two independent experiments.** b** Protein amounts from cultivation of M44 transformants with deletion of *crt1* or overexpression of various transporters in minimal lactose medium with two different lactose concentrations. Error bars present standard deviation between growth replicates (*n* = 2)

Since the *crt1* gene had been deleted and overexpressed in literature in *T. reesei* strain QM9414, which is an early cellulase-producing mutant, we wanted to see what the effect of the same modifications would be in a better production strain. M44 is a strain derived from QM9414 which has gone through additional rounds of mutagenesis to improve its protein production, but it suffers from a protein secretion defect when grown on minimal lactose medium. We looked first into differences between QM9414, M44 and an additional strain, RUT-C30, which is a carbon catabolite derepressed cellulase hyperproducer mutant strain. Analysis of protein secreted into supernatant during cultivation of these three strains on minimal medium with either glucose, cellobiose or lactose as carbon source revealed large differences between the strains (Fig. 3a). While M44 produced the highest amount of protein on cellobiose, RUT-C30 had the highest protein production on lactose where also QM9414 secreted slightly more protein than M44, due to the aforementioned protein secretion defect of the latter strain. Compared to the two other strains, M44 also grew to lower final cell density on both cellobiose and lactose while on glucose medium the differences were smaller between the strains (Additional file 1: Figure S1A).

Analysis of the expression of the known cellulase regulating transcription factor *xyr1*, *crt1* and one of the main cellulase genes, cellobiohydrolase *cbh1*, during growth of these strains on minimal medium with different carbon sources also revealed differences between the strains (Additional file 1: Figure S1B). As expected, expression levels of all these genes were higher on cellobiose or lactose medium than in glucose medium in all of the strains, although in some cases this was only evident in the later timepoint. In the carbon catabolite derepressed RUT-C30 strain, smaller differences in the expression levels were seen between inducing and non-inducing conditions based on log$_{2}$ fold-changes of the expression levels than in the other two strains. This was especially true for *crt1*, for which significantly lower fold-changes were measured in the RUT-C30 strain in the later timepoint compared to the two other strains (*t*-test: *p* < 0.05). In M44, all tested genes were induced by both cellobiose and lactose in both timepoints, while in QM9414 *cbh1* and *crt1* were induced later on cellobiose than on lactose. However, in QM9414 the expression levels of these genes were lower than in the other two strains. Since all the studied genes were clearly induced in M44 on both cellobiose and lactose to similar expression levels, their expression was not likely the cause of the observed protein secretion defect.

Since CRT1 had been published as a lactose transporter, we decided to investigate how its deletion and overexpression in M44 would affect the protein secretion of this strain in minimal lactose medium. We chose both RUT-C30 and QM6a versions of CRT1 for overexpression studies, as well as two heterologous fungal sugar transporters (*N. crassa* CDT-1 and *A. nidulans* LacpB/CltB) which had been shown to be able to transport cellobiose when expressed in yeast [8, 10]. LacpB has also been shown to influence lactose metabolism of *A. nidulans* [17]. Expression cassettes were constructed where the different genes were expressed under the constitutive *pdc1* promoter and they were integrated into the *pep1* locus of the strain M44.

When the subsequent strains were cultured, it was discovered that the lactose related protein secretion defect of M44 was rescued by the expression of the CRT1-RUT-C30 or by the expression of either of the heterologous transporters, with CDT-1 expression being most beneficial for total protein production (Fig. 3b). Expression of the CRT1-QM6a also seemed to improve protein production of the strain on minimal medium with 2% lactose, but not as much on 1% lactose. As expected, $\Delta$*crt1* strain did not secrete protein when grown on lactose medium.

### Deletion of *crt1* can be complemented by the expression of heterologous transporters

**Fig. 4:**
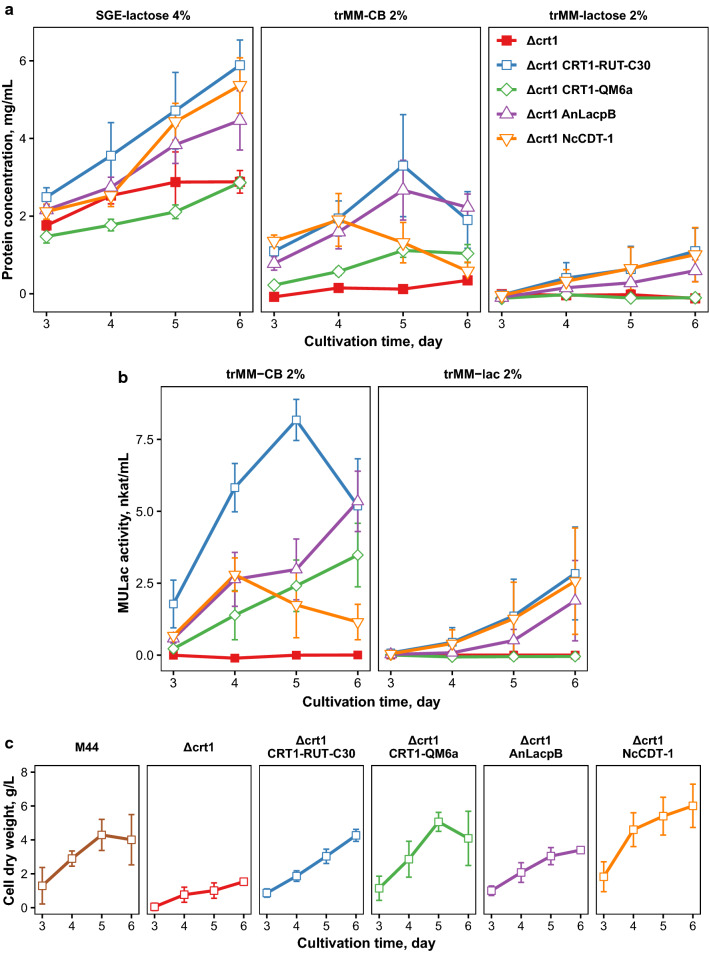
Complementation of *crt1* deletion by overexpression of the RUT-C30 version of CRT1 or by heterologous transporters.** a** Protein secreted into medium by $\Delta$*crt1* strain expressing different transporters and grown in minimal medium with indicated carbon sources.** b** Cellulase activities from cultivation of the same strains, measured as methylumbelliferyl-$\upbeta$-d-lactoside (MULac) hydrolysis. Colors and symbols are same as in** a**. Error bars in** a**,** b** present standard deviation between 2 growth replicates of two biological replicates (*n* = 4).** c** Cell dry weights of the strains with trMM-CB 2% as carbon source. Error bars present standard deviation between three independent experiments

Prior studies have suggested that CRT1 may function as a lactose/cellulose sensor. In order to address this possibility, we did complementation studies in the *crt1* deletion strain. We wanted to investigate which of the transporters could complement the $\Delta$*crt1* phenotype and therefore the expression constructs with constitutive *pdc1* promoter were transformed to the *crt1* deletion strain. Interestingly, expression of the CRT1-RUT-C30 or either of the heterologous permeases complemented *crt1* deletion, as revealed by protein concentration and cellulase activity measurements from the cultivation supernatant (Fig. 4a, b). As seen from Fig. 4a, the effect was seen also on complex medium, which contained spent grain extract and lactose. These results were supported by cell dry weight measurements during cultivation on cellobiose, which also indicated that the strain expressing CDT-1 grows faster than the other strains on this carbon source (Fig. 4c). The results further indicated that the $\Delta$*crt1* strain grows slower on cellobiose than the other tested strains (*t*-test: *p* < 0.05 for all strains on at least one timepoint). Interestingly, the expression of CRT1-QM6a also complemented the growth defect of the $\Delta$*crt1* deletion strain on cellobiose and allowed some protein secretion on this carbon source. However, its expression did not rescue the protein secretion defect on lactose, as was the case for the strain where the genomic copy of *crt1* was intact. We also did HPLC measurements which revealed that cellobiose consumption was faster in all of the complementation strains than in the $\Delta$*crt1* strain (Additional file 1: Figure S2). Sugar consumption was especially increased by the expression of CDT-1, as observed in both wild-type and $\Delta$*crt1* backgrounds. In the *crt1* deletion background, it is important to point out that expression of *N. crassa* CDT-1 or *A. niger* LacpB provided the strain with the ability to secrete enzymes. In this respect, the general effect was the same whether a heterologous transporter or CRT1-RUT-C30 was expressed. This would suggest that the flow of cellobiose or lactose into the cell is the main critical factor in triggering enzyme induction. It was best to introduce the CRT1-RUT-C30, which gave the highest total protein and cellulase activity values. Surprisingly, CDT-1 expression did not provide the highest cellulase activities or total protein values. Instead, expression of CDT-1 seemed to result in a diminishing effect over time, which may be linked to its high disaccharide uptake rate since it has previously been observed that *T. reesei* produces highest amount of protein at low growth rates [27].

**Fig. 5:**
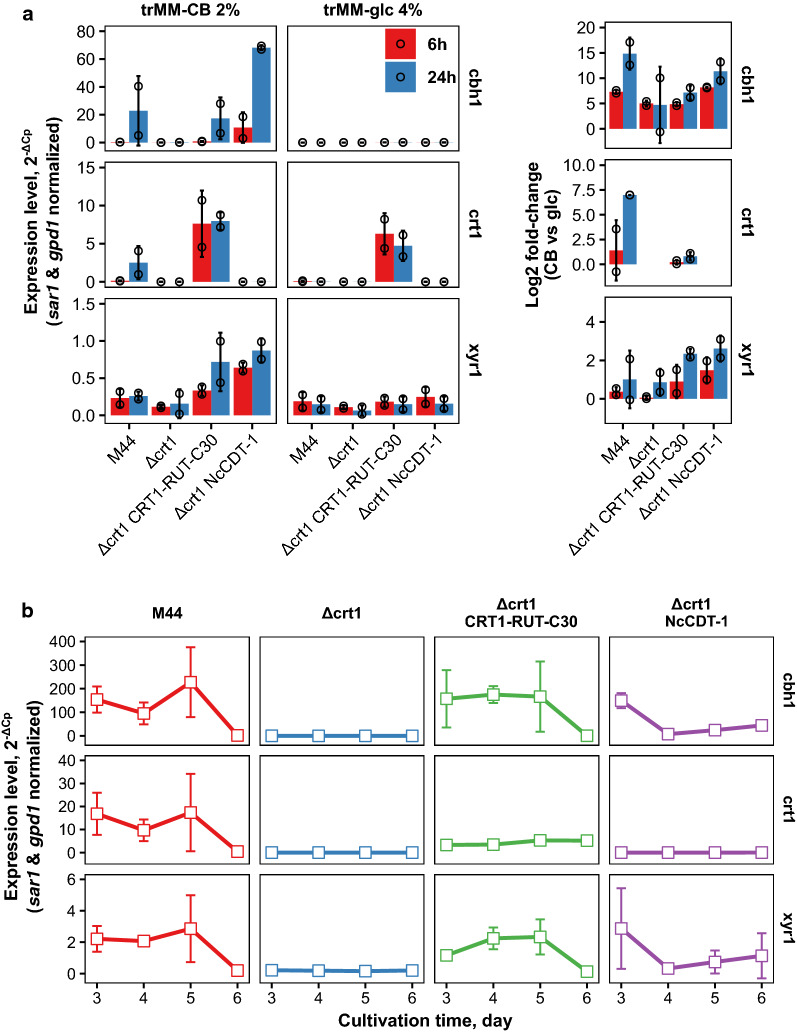
Gene expression analysis of* T. reesei* $\Delta$ crt1 complementation strains.** a** Expression levels after medium switch from neutral (glycerol) to inducing or repressing conditions (trMM-2% CB or trMM-4% glc, respectively). Both expression levels and fold-changes (inducing vs non-inducing) are shown. Fold-changes of *crt1* expression were set to zero for *crt1* deletion strains for the sake of clarity. Error bars present standard deviation between two independent experiments.** b** Expression levels during cultivation in trMM-2% CB. Error bars present standard deviation between two independent experiments

To further gain insight into CRT1 complementation by CRT1 itself or by CDT-1, we analyzed gene expression with RT-qPCR. First we did an experiment where washed mycelia was inoculated into cellobiose or glucose medium and the expression of selected genes was analyzed 6 h and 24 h after the inoculation (Fig. 5a). As expected, *crt1* expression was detected in cellobiose medium in the parental M44 strain and in both media in the complementation strain where CRT1-RUT-C30 was expressed with the constitutive *pdc1* promoter, but absent from the *crt1* deletion strain and from the strain where *crt1* deletion was complemented by CDT-1 expression. Expression of cellobiohydrolase *cbh1* was only detected in the parental strain M44 or in the strains where the deletion of *crt1* was complemented by the expression of CRT1-RUT-C30 or by CDT-1. *Xyr1* expression was detected in all strains in both carbon sources, but it was clearly induced by cellobiose in the complementation strains. The expression of another cellulase regulator, *ace3*, was also analyzed and the results were similar to that of *xyr1*: although the expression levels of *ace3* were lower, the fold-changes were in the same range as with *xyr1* (data not shown). Interestingly, expression of both *xyr1* and *ace3* were also detected in the deletion strain which has a defective induction system.

To see how the gene expression varied in normal cultivation conditions, total RNA was extracted on days 3–6 from the same strains grown on minimal cellobiose medium (Fig. 5b). As seen from the figure, the deletion strain clearly had very low expression levels for all of the tested genes during the experiment. No large differences were seen between wild-type and CRT1-RUT-C30 expressing strain, except that the *crt1* expression was higher in the wild-type strain due to higher strength of the native *crt1* promoter as opposed to constitutive *pdc1* promoter which was used in the complementation strain. Although *crt1* expression was lower in the complementation strain which expressed CRT1-RUT-C30 than in the wild-type strain, similar levels of *cbh1* and *xyr1* expression were detected during the cultivation. Importantly, the expression of CDT-1 also triggered *cbh1* and *xyr1* expression. However, in the strain which expressed CDT-1, *cbh1* and *xyr1* were expressed earlier in the cultivation, probably due to higher rate of sugar uptake and faster growth.

### CRT1 manipulation in QM9414 background

**Fig. 6:**
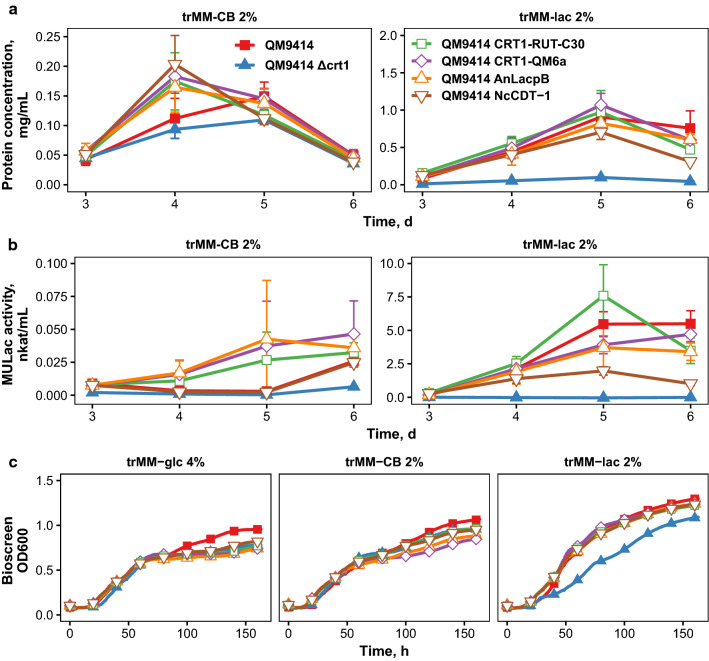
Overexpression of CRT1 or heterologous transporters in the QM9414 background.** a** Protein concentrations of the cultivation supernatant on minimal cellobiose or lactose medium. Error bars represent standard deviation between four growth replicates.** b** MULac activities of the cultivation supernatant, error bars as in** a**.** b** Growth curves on cellobiose and lactose medium, measured with the Bioscreen C incubator. Colors and symbols for all plots are described in** a**

Since our results were obtained in a different background than other published work about CRT1, we decided to repeat the modifications in QM9414. Overexpression of both versions of *crt1* or of the heterologous transporters seemed to improve protein production on cellobiose on day 4, but the differences were small and on lactose no differences were seen. The $\Delta$*crt1* strain had lower protein production on lactose as expected (Fig. 6a). When compared to M44-based strains, the lower protein production on cellobiose as compared to lactose was remarkable. However, the QM9414 strain seems to grow much better in lactose than M44 as was already shown in Additional file 1: Figure S1A. The deletion mutant seemed to have no cellulase activity even on cellobiose (Fig. 6b). This is not in agreement with published literature, but in the previous studies, the inoculation method was different (spores in this study, mycelium in the other studies), which may affect the results [13, 14]. Expression of either version of CRT1 or AnLacpB seemed to increase cellulase activities on cellobiose, although the activities were very small when compared to those obtained on lactose medium. Interestingly, although the amount of protein secreted by the strain expressing CDT-1 was similar to that of the other strains, the cellulase activities were lower for this strain and similar to that of the parental strain on cellobiose. On lactose medium, the strain expressing CDT-1 produced less cellulases than the parental strain (*t*-test: *p* < 0.01 on days 5 and 6). Growth profiling with Bioscreen C verified the faster onset of growth of the strains overexpressing any of the transporters on cellobiose and lactose medium, which had also been predicted from the protein measurements (Fig. 6c).

In literature there has been conflicting views about the effect of *crt1* deletion on induction by sophorose [13, 14]. To test this, we added sophorose to cultures of M44 and QM9414-based strains which had been grown on sorbitol for 3 days and measured protein concentrations for 4 days after the addition (Additional file 1: Figure S3). As seen from the figure, the effect of sophorose addition to protein concentration was much larger in M44 background. It was observed that the addition had no effect on the protein produced by the deletion strain, but the parental strain and the strains expressing CRT1-RUT-C30 or CDT-1 produced more protein after the addition of sophorose (*t*-test: *p* < 0.05 for $\Delta$*crt1* vs other strains after day 4). In the QM9414 background the differences were negligible between the strains.

## Discussion

The uptake of sugars is crucial for the growth and is intricately linked to cellulase and hemicellulase production by *T. reesei*. Secreted enzyme mixtures break down complex carbohydrates in its environment and release smaller transportable sugars that can be taken up by the fungus. In contrast to *A. nidulans* or *N. crassa*, deletion of only one sugar uptake transporter seems to cause the lack of cellulase induction on multiple substrates in *T. reesei*. Functional testing of CRT1 with the yeast system revealed that while initially characterized as a lactose permease, this transporter prefers cellobiose, although it has high affinity for both of these disaccharides. Many transporters from filamentous fungi seem to have very high affinities for their substrates, which probably reflects their natural habitat where sugars are scarce. In contrast to saprotrophic fungi, yeast *S. cerevisiae* has evolved to grow, for example, on grape juices with sugar concentrations in range of hundreds of grams per liter of glucose and fructose and as a result does not possess sugar transporters which such high affinities. *S. cerevisiae* transporters responsible for glucose transport (HXT1–7) can be divided into low ($K_{\mathrm{m}}$ 100–200 mM), moderate (10–15 mM) or high-affinity transporters (1–3 mM) [28]. The two high-affinity glucose transporters identified from *N. crassa* by Wang et al. [18], HGT-1 and HGT-2, have $K_{\mathrm{m}}$ values of 16 and 99 $\upmu$M, respectively. Glucose transporters that have thus far been kinetically characterized from *T. reesei* also have low $K_{\mathrm{m}}$ values for glucose, 10–60 $\upmu$M [4]. The results obtained in this study indicate that CRT1 is also a high-affinity transporter for its substrates cellobiose and lactose. The kinetics results obtained for CRT1 were similar to those obtained for *N. crassa* CDT-1 regarding both cellobiose and lactose. Both proteins also share similar mechanism with the transport of sugar coupled to transport of protons.

Analysis of protein production, growth and gene expression in the three mutant strains, M44, QM9414 and RUT-C30, revealed differences between the strains. Gene expression analysis indicated that in the RUT-C30 strain *crt1* is expressed also in the glucose medium early in the cultivation, while the expression levels of *cbh1* and *xyr1* were almost nonexistent on glucose in the first timepoint. Interestingly, protein production also started earlier in RUT-C30 than in QM9414 on lactose. This would suggest that the higher basal expression of *crt1* might be beneficial for the onset of cellulase production. In strain M44, the protein production and gene expression results were different between the two inducing media; when this mutant strain was grown on lactose medium, only low levels of protein were secreted although all the studied genes were clearly induced at the studied time points. This phenotype was rescued by the constitutive expression of CRT1-RUT-C30, or the heterologous cellodextrin transporters, which would indicate that the defect was caused by lactose transport. The expression of CRT1-QM6a seemed to also improve protein production on lactose, although the effect was smaller and dependent on lactose concentration, unlike the effect seen for the other expressed transporters. In the $\Delta$*crt1* background, only the expression of heterologous transporters or CRT1-RUT-C30 rescued the cellulase induction defect on both lactose and cellobiose medium, while the expression of the CRT1-QM6a enabled some protein production on cellobiose medium. Gene expression analysis of the M44-based strains where the *crt1* deletion was complemented by the expression of CRT1-RUT-C30 or CDT-1 revealed that transcription factors *xyr1* and *ace3* were more upregulated in the complementation strains than in the wild-type M44 after shift to inducing conditions. This supports the aforementioned hypothesis that the onset of cellulase production can be improved by the constitutive expression of a transporter which is capable of transporting the inducer (in this case cellobiose or lactose). When overexpressed in QM9414, both CRT1 versions and both of the heterologous transporters seemed to hasten the onset of protein production on cellobiose. The results about earlier onset of cellulase induction in a strain overexpressing CRT1 are in line with the results obtained by Ivanova et al. [14].

Interestingly, although CRT1-QM6a did not seem to have any transport activity in yeast, it seemed to possess the ability to partially complement the protein secretion defect of the M44 strain on lactose, and to some extent rescue the cellulase induction defect of the $\Delta$*crt1* strain in medium containing cellobiose as the carbon source. Also, although the $\Delta$*crt1* strain expressing CRT1-QM6a did not produce as much protein on cellobiose medium than the one expressing CRT1-RUT-C30, the growth curves derived from cell dry weight measurements were similar to that of the parental strain. Since the differences in annotation concern only the N-terminal intracellular part of the protein, it might be that the CRT1-QM6a also has some minimal transport activity. This QM6a version (Trire2_3405) was used in the previously mentioned reports about CRT1, which might explain why no uptake was seen in the yeast experiments [13, 14].

The importance of the N- and C-termini of sugar transport proteins has been shown, e.g., by mutagenesis studies by Zhang et al. [29]. In that study the switching of intracellular N- and C-terminal segments of *N. crassa* HGT-1 to replace the native segments of *T. reesei* STP1 allowed this transporter to confer almost as good growth of yeast as *HGT-1* itself without losing the cellobiose transport activity. The authors also discovered that the truncation of the C-terminal tail of STP1 by 16 amino acids improved growth significantly on glucose, while no growth was seen on glucose if the truncation was extended to 32 amino acids. The intracellular parts have also been shown to be involved in substrate-induced ubiquitination and subsequent degradation of *N. crassa* cellodextrin transporters when heterologously expressed in yeast [30]. Analysis of the CRT1 amino acid sequence by UbPred ubiquitination site prediction server [31] gave two possible ubiquitination sites, both located in the N-terminal intracellular part of the protein. The first site (residue 4, predictor score 0.83, medium confidence) resides in the part that is missing from CRT1-QM6a, but the second one (residue 35, score 0.62, low confidence) is present in both versions. Recently, Mikros and Diallinas [32] reviewed the evidence of the functionality of the N- and C-termini of yeast and fungal transporters and concluded that they can have effects, e.g., on transport activity, substrate specificity, membrane insertion and recycling. The results obtained for the N-terminally truncated CRT1-QM6a might indeed be explained by a change in substrate specificity or affinity when compared to the longer and more active CRT1-RUT-C30.

In a way, the result seen in our study for CRT1-QM6a shows that a membrane protein with no or only minimal transport activity can still rescue the cellulase induction defect. This observation is similar to results obtained by Znameroski et al. [9] where *N. crassa* CDT-1 and CDT-2 were shown to behave in a similar way. One possible interpretation may be that these proteins can function as transceptors with signaling ability or that the intracellular system for detection of the inducer has very high affinity and gets activated with very low concentrations of the inducer. In this study, the expression of heterologous transport proteins CDT-1 and LacpB in a strain containing a deletion of the *crt1* gene was able to rescue the induction of enzyme secretion. This is an interesting observation, because this suggests that there is not necessarily anything unique about CRT1 that would be required to trigger enzyme induction. One explanation is that simply moving cellobiose or lactose inside the fungal cell triggers the induction cascade, although this does not exclude the possibility of CRT1 having signaling function. Likewise it has been suggested that the *N. crassa* cellodextrin transporters CDT-1 and CDT-2 could function as signaling transceptors, although it is unknown if that mechanism would also function heterologously in *T. reesei*. It is unknown if AnLacpB also may function as a transceptor, but it has been reported be a disaccharide transporter [10, 17]. In our studies, expression of heterologous transporter, CDT-1, in the $\Delta$*crt1* strain led to the induction of the *ace3*, *xyr1* and *cbh1* genes similarly to what happened after CRT1 was re-expressed in the strain. Both ACE3 and XYR1 transcription factors are indispensable for the cellulase induction [23, 33]. In a recent study by Zhang et al. [34], ACE3 was shown to bind to the promoter of *crt1* gene and deletion of *ace3* resulted in downregulation of *crt1* expression in strain QM6a (log$_{2}$ fold-change − 10.07 between $\Delta$*ace3* and wild-type). According to the authors, *crt1* promoter also contains binding sites for XYR1.

**Fig. 7:**
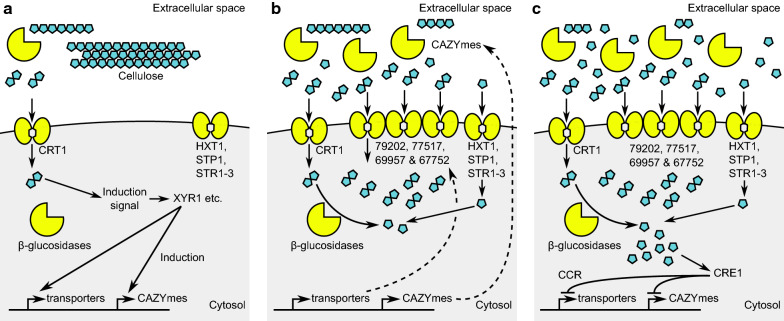
Hypothesis about the role of CRT1 in cellulase induction.** a** Scouting activity of cellulases results in the formation of small amount of inducing sugar (cellobiose or sophorose). The sugar is imported by CRT1 and sensed by some unknown component of the induction system with high affinity for the inducer. Binding of the inducer to this component causes the cellulase induction.** b** Among cellulases, lower affinity cellobiose transporters and other transporters (arabinose, xylose, etc.) get induced, resulting in the transport of bulk of the sugars released from the lignocellulose feedstock.** c** Accumulation of glucose inside the cells by $\upbeta$-glucosidase activity and by the scouting high-affinity hexose transporters causes the carbon catabolite repression to limit cellulase production

Since CRT1 is a high-affinity cellobiose transporter it remains to be elucidated what are the affinities and roles of the many other disaccharide transporters of *T. reesei*. After all, it might be that once CRT1 initiates the cellulase induction cascade by transporting a very small amount of cellobiose released from cellulose, the induction signal caused by some unknown factor also causes the other transporters to be induced as sketched in Fig. 7a. After their production is induced, transporters are translocated to the cell membrane and cellulases are secreted into the medium, resulting in higher rates of mono- and disaccharide uptake (Fig. 7b). The glucose which has been uptaken by glucose transporters and released from cellobiose by intracellular $\upbeta$-glucosidases accumulates inside the cell in sufficient concentration to trigger the carbon catabolite repression via CRE1 (Fig. 7c). The previously mentioned disaccharide transporters might include the two putative lactose transporters identified by Porciuncula et al. [15] (trire2_79202 and 77517) and the two recently identified transporters which both have been shown to have cellobiose transport activity when expressed in yeast (trire2_69957 and 67752) [25, 35]. The genes coding for these transporters are expressed in cellulase-inducing conditions. However, this proposed scouting role of CRT1 is not fully supported by its expression profile since it is upregulated by its seemingly preferred substrate cellobiose, instead of being repressed by it as is the case for many other high-affinity transporters (e.g., yeast *HXT6* and *HXT7*, *N. crassa* *HGT-1* and *HGT-2*). The expression of *crt1* seems to be be controlled by *xyr1*, *ace3* and also by *cre1* [34, 36, 37]. The *N. crassa* cellodextrin transporters CDT-1 and CDT-2 are also upregulated in cellulase-inducing conditions and they are differentially expressed in deletion strains of cellulase regulators CLR-1 and CLR-2 and CDT-2 also in XLR-1 deletion strain [38]. Recent analysis indicates that both are also directly regulated by CRE-1 [39].

Studies done by Kubicek et al. [40] support the presence of a high-affinity cellobiose transporter in *T. reesei*. In the study a system for cellobiose uptake was discovered with a $K_{\mathrm{m}}$ value of 0.3 $\upmu$M from glucose-grown mycelia from QM9414. This system, which was shown to act via proton symport mechanism, was shown to be inhibited by gentiobiose, laminaribiose, sophorose and glucose, but not by lactose. In the same study the authors also observed that the permease was inducible by sophorose, and that its induction preceded that of *cbh1*. No induction by cellobiose was seen in the timeframe of the experiment (180 min), although induction by sophorose was seen already about 30 min after it was added to the medium [40]. In an earlier study by Sternberg and Mandels [41] a low affinity uptake system for sophorose was also described, and in the same study a cellobiose uptake activity was described with the same pH optimum as in the study by Kubicek et al. [40].

A cellobiose transporter must have an important role in cellulase induction, as this sugar has been shown to also cause cellulase induction in *T. reesei*. The potential of cellobiose as inducer of cellulase production was demonstrated as early as 1990 by Fritscher et al. [42], who came to the conclusion that cellobiose acts as an inducer in conditions that favor its uptake rather than its extracellular hydrolysis to glucose, a repressing carbon source. The authors also speculated that the reason for the superiority of sophorose as inducer could be related to its low hydrolysis by $\upbeta$-glucosidases. In a more recent article, Jourdier et al. [43] demonstrated that cellobiose acts as a inducer in fed-batch cultivation under carbon flux limitation in a carbon catabolite derepressed strain. It might be that under carbon limitation the high-affinity uptake system for cellobiose is more active than extracellular hydrolysis, especially since the extracellular $\upbeta$-glucosidases of *T. reesei* have lower affinity for cellobiose than CRT1 ($K_{\mathrm{m}}$ values 0.37, 0.31 and 0.79 mM for cel3A, cel3B and cel3E, respectively) [44].

Protein production by *T. reesei* has also been shown to be higher under low growth rates in studies by Pakula et al. [27]. Also in our experiments, it seemed that increased growth rate conferred by CDT-1 expression had negative impact on protein production. The expression of CDT-1 seemed to lead to transport of substrates faster compared to CRT1, which can be seen by the growth rates and sugar consumption measured for the strains. Thus, the cellobiose uptake rate mediated by CRT1 seems to provide optimal substrate uptake suitable for this organism’s carbon metabolism.

## Conclusions

In this study CRT1 was functionally characterized to be a high-affinity cellobiose transporter that also has the ability to transport lactose. Analysis of *crt1* expression in different *T. reesei* strains revealed that in RUT-C30, *crt1* expression is higher in non-inducing conditions than in M44 or QM9414, which might explain the faster initiation of protein production in lactose medium by this strain. Manipulation of *crt1* expression revealed that its overexpression removed the protein secretion defect of M44 on minimal lactose medium, and resulted in earlier initiation of cellulase production in QM9414 when grown on cellobiose. Deletion of *crt1* could be complemented by CRT1-RUT-C30 and also by heterologous cellodextrin transporters from *N. crassa* and *A. nidulans*. Partial complementation of the cellulase induction defect was observed also when CRT1-QM6a was expressed in the $\Delta$*crt1* strain, but this effect was seen only on cellobiose medium, and not in lactose medium. The faster growth conferred by the expression of *N. crassa* CDT-1 seemed to have negative effect on protein secretion. The results are in line with the role of CRT1 as a high-affinity cellobiose transporter, which can transport the soluble cellulase inducer into the cell where it is sensed by some other component of the induction system which then sends the induction signal.

## Methods

### Microbial media, cultivation and chemicals

*T. reesei*, *A. nidulans* and *N. crassa* strains were routinely cultivated on potato dextrose agar (PDA). Uridine-auxotrophic mutants of *T. reesei* strains QM9414 and M44 were used as hosts for genetic engineering with *pyr4* selection. Minimal medium for* T. reesei* consisted of 15 g/L KH$$_{2}$$PO$$_{4}$$, 5 g/L (NH$$_{4}$$)$$_{2}$$SO$$_{4}$$, 1 mL/L trace element stock (FeSO$$_{4}$$$$\cdot$$7H$$_{2}$$O 5 g/L, MnSO$$_{4}$$$$\cdot$$H$$_{2}$$O 1.6 g/L, ZnSO$$_{4}$$$$\cdot$$7H$$_{2}$$O 1.4 g/L, CoCl$$_{2}$$$$\cdot$$6H$$_{2}$$O 3.7 g/L) and indicated carbon source. SGE-lactose medium consisted of minimal medium supplemented with 40 g/L lactose, 20 g/L spent grain extract, 100 mM PIPPS and 8.6 g/L di-ammonium citrate, and with ammonium sulfate replaced with 5.4 g/L NaSO$$_{4}$$. For looping out the *pyr4* marker, the media was supplemented with 1.5 g/L 5-FOA and 5 mM uridine. Liquid media was adjusted to pH 4.8, autoclaved and afterwards MgSO$$_{4}$$ and CaCl$$_{2}$$ were added to 2.4 mM and 4.1 mM final concentrations, respectively. Solid media contained 20 g/L agar and was supplemented with Triton X-100 as appropriate to restrict the spread of the mycelium. *T. reesei* spore suspensions were prepared by collecting spores from a PDA plate into a solution which contained 0.8% NaCl, 0.025% Tween 20 and 20% glycerol and the resulting suspensions were stored in − 80 $$^\circ \hbox { C}$$. In yeast studies, synthetic complete (SC) -Ura or -Ura-His medium was used with 2% glucose (CEN.PK2-1D background) or 2% glycerol and 2% ethanol (AFY09 background) as carbon sources, unless otherwise indicated.

For the *T. reesei* cultivations, 2$\cdot$10$^{5}$ spores/mL were inoculated to 4 mL of medium in 24-well deep-well microtiter plates (MTP) and the plates were incubated in 28 $^\circ \hbox { C}$ with 800 rpm shaking (3 mm throw) in Infors HT microtron incubator (Bottmingen, Switzerland). For the RT-qPCR experiment where the mycelium was washed before inoculation into inducing or non-inducing conditions, precultures were grown in shake flasks with 50 mL medium in 250 mL baffled shake flasks for 4 days in 28 $^\circ \hbox { C}$ and 200 rpm shaking. Yeast was also cultured either on 24- or 96-well deep-well microtiter plates (28 $^\circ \hbox { C}$, 800 rpm, 4/1 mL medium) or in non-baffled shake flasks (50 mL in 250 mL) in 30 $^\circ \hbox { C}$ and 230 rpm.

Chemicals were obtained from Sigma-Aldrich (Saint Louis, MO, USA) and molecular biology reagents from Thermo Fisher Scientific (Waltham, MA, USA) unless otherwise mentioned. Lactose was obtained from VWR (Helsinki, Finland) and $\upalpha$-sophorose from Serva Electrophoresis GmbH (Heidelberg, Germany). Strain AFY09 was a gift from Prof. Dr. Eckhard Boles from Goethe University and strain yJR33 was provided by Dr. Jorg de Ruijter from VTT Technical Research Center of Finland.

### Molecular biology techniques

*Escherichia coli* and *S. cerevisiae* colony PCR was done with DreamTaq polymerase and *T. reesei* colonies were screened with Phire Plant kit. High-fidelity PCR was done with Kapa Hifi polymerase (Roche, Basel, Switzerland). Oligonucleotides were ordered from Sigma-Aldrich and synthetic genes from Thermo Fisher Scientific. Plasmid DNA was prepared with GeneJet miniprep kit and gel extractions and PCR purifications with respective Qiaquick kits (Qiagen, Hilden, Germany). *T. reesei* gDNA was prepared by Easy-DNA kit, and *N. crassa* and *A. nidulans* gDNA with phenol-chloroform extraction. Assembly of MoClo plasmids and Golden gate cloning was done as described by Lee et al. [45]. Yeast transformation was done by the method by Gietz and Woods [46]. Yeast homologous recombination was done by transforming overlapping fragments into yeast strain FY834 and by rescuing the plasmid from the resultant colonies [47, 48]. *T. reesei* transformation was done as described by Penttilä et al. [49].

**Table 1:** Strains constructed in this study

Name	Description/genotype
*Aspergillus nidulans*	
FGSC A4	WT strain
*Neurospora crassa*	
FGSC 9719	WT strain
*Trichoderma reesei*	
QM9414	Early producer strain with improved protein production
M44	Mutated QM9414
RUT-C30	Carbon catabolite derepressed mutant strain
M44 $\Delta$*crt1*	M44 $\Delta$*crt1*
trSS-1	M44 *pep1*::*crt1*-RUT-C30 (P$_{\mathrm{pdc1}}$)
trSS-2	M44 *pep1*::*crt1*-QM6a (P$_{\mathrm{pdc1}}$)
trSS-6	M44 *pep1*::*AnLacpB* (P$_{\mathrm{pdc1}}$)
trSS-7	M44 *pep1*::*NcCDT-1* (P$_{\mathrm{pdc1}}$)
trSS-8	M44 $\Delta$*crt1* *pep1*::*crt1*-RUT-C30 (P$_{\mathrm{pdc1}}$)
trSS-9	M44 $\Delta$*crt1* *pep1*::*crt1*-QM6a (P$_{\mathrm{pdc1}}$)
trSS-12	M44 $\Delta$*crt1* *pep1*::*AnLacpB* (P$_{\mathrm{pdc1}}$)
trSS-13	M44 $\Delta$*crt1* *pep1*::*NcCDT-1* (P$_{\mathrm{pdc1}}$)
trSS-14	QM9414 $\Delta$*crt1*
trSS-15	QM9414 *pep1*::*crt1*-RUT-C30 (P$_{\mathrm{pdc1}}$)
trSS-16	QM9414 *pep1*::*crt1*-QM6a (P$_{\mathrm{pdc1}}$)
trSS-18	QM9414 *pep1*::*AnLacpB* (P$_{\mathrm{pdc1}}$)
trSS-19	QM9414 *pep1*::*NcCDT-1* (P$_{\mathrm{pdc1}}$)
*Saccharomyces cerevisiae*	
FY834	MAT$\upalpha$ his3$\Delta$200 ura3-52 leu2$\Delta$1 lys2$\Delta$202 trp1$\Delta$63
AFY09	EBY.VW5000 mal31$\Delta$::loxP-kanMX4-loxP mal21$\Delta$::loxP-hphNT1-loxP
CEN.PK2-1D	MAT$\upalpha$ ura3-52 his3-$\Delta$1 leu2-3,112 trp1-289 MAL2-8$^{\mathrm{c}}$
ySS1	AFY09 *URA3*::*NcGH1-1* (P$_{\mathrm{PGK1}}$, T$_{\mathrm{ADH1}}$, *HIS3*)
ySS1 CRT1-RUT-C30	ySS1 crt1-RUT-C30 (P$_{\mathrm{PGK1}}$, T$_{\mathrm{ENO1}}$, *CEN6*/*ARS4*, *URA3*)
ySS1 CRT1-QM6a	ySS1 crt1-QM6a (P$_{\mathrm{PGK1}}$, T$_{\mathrm{ENO1}}$, *CEN6*/*ARS4*, *URA3*)
ySS1 NcCDT-1	ySS1 NcCDT-1 (P$_{\mathrm{PGK1}}$, T$_{\mathrm{ENO1}}$, *CEN6*/*ARS4*, *URA3*)
ySS1 NcCDT-2	ySS1 NcCDT-2 (P$_{\mathrm{PGK1}}$, T$_{\mathrm{ENO1}}$, *CEN6*/*ARS4*, *URA3*)
ySS1 CEL1a	ySS1 cel1a (P$_{\mathrm{PGK1}}$, T$_{\mathrm{ENO1}}$, *CEN6*/*ARS4*, *URA3*)
yJR33	CEN.PK2-1D *URA3*::*NcGH1-1* (P$_{\mathrm{PGK1}}$, T$_{\mathrm{ADH1}}$, *HIS3*)
yJR33 CRT1-RUT-C30	yJR33 crt1-RUT-C30 (P$_{\mathrm{PGK1}}$, T$_{\mathrm{ENO1}}$, *CEN6*/*ARS4*, *URA3*)
yJR33 CRT1-QM6a	yJR33 crt1-QM6a (P$_{\mathrm{PGK1}}$, T$_{\mathrm{ENO1}}$, *CEN6*/*ARS4*, *URA3*)
yJR33 NcCDT-1	yJR33 NcCDT-1 (P$_{\mathrm{PGK1}}$, T$_{\mathrm{ENO1}}$, *CEN6*/*ARS4*, *URA3*)
yJR33 NcCDT-2	yJR33 NcCDT-2 (P$_{\mathrm{PGK1}}$, T$_{\mathrm{ENO1}}$, *CEN6*/*ARS4*, *URA3*)
yJR33 CEL1a	yJR33 cel1a (P$_{\mathrm{PGK1}}$, T$_{\mathrm{ENO1}}$, *CEN6*/*ARS4*, *URA3*)

### Strain and plasmid construction

Strains used in this work are listed in Table 1 and primers in Additional file 2: Table S1. For heterologous expression of fungal transporters in yeast, the transporters were ligated into a MoClo expression vector with *PGK1* promoter, *ENO1* terminator, *CEN6*/*ARS4* replication and *URA3* selection (MoClo parts pYTK-2, -11, -51, -67, -74, -81, -84; [45]). For testing of yeast strains for growth on cellobiose or lactose, codon-optimized *N. crassa* $\upbeta$-glucosidase *GH1-1* gene was expressed with a yeast expression vector made with the MoClo system (parts pYTK-2, -11, -53, -67, -76, -82, -83). The expression cassette containing *GH1-1* with *PGK1* promoter, *ADH1* terminator and *HIS3* marker was amplified with *URA3* flanks with primers SaSS-25 and -27 and transformed into yeast for integration into *URA3* locus. Correct integration of the cassette and absence of the *URA3* ORF was verified with PCR with primer pairs oSK-192 and SaSS-17, oSK-193 and SaSS-18, as well as oSK-186 and oSK-187. The modifications were done in hexose-positive (strain yJR33) and hexose-negative (strain ySS1) strain backgrounds.

*Trichoderma reesei* gene *crt1* (Trire2_3405) was expressed with two sequences differing on the N-terminus due to different versions being present in genome annotations of *T. reesei* QM6a and RUT-C30 [19, 20]. The amino acid sequences for the QM6a and RUT-C30 versions are available from GenBank with Accession numbers EGR49861.1 and ETS03552.1, respectively [50]. Both versions of the genes were amplified with primers SaSS-30–34 from *T. reesei* gDNA and ligated to *T. reesei* expression vector B6243 with yeast homologous recombination. The vector contains *pdc1* promoter, *pyr4* as selection marker, *cbh1* terminator, pRS426 backbone [51] and is targeted for integration into *pep1* locus. For the expression of heterologous disaccharide permeases *AnLacpB* and *NcCDT-1*, the ORFs were amplified with primers SaSS-49–52 from gDNA of *A. nidulans* or *N. crassa* and ligated to B6243 with yeast homologous recombination. The expression plasmids were digested with *Pme*I and the fragment containing the expression cassette for the gene and *pyr4* marker was gel purified before transformation into *T. reesei*.

For the deletion of *crt1* gene, about 1000 bp fragments of the 5′ and 3′ regions surrounding the QM6a version of the gene were amplified with primers C132–C134. The primers contained about 30 bp of overlapping sequence with pRS426 backbone and the amdS expression cassette from pTTv249 [52]. pRS426 backbone was digested with *Eco*RI and *Xho*I and gel purified. The amdS expression cassette was liberated from pTTv249 with *Not*I digestion and gel purified. The two amplicons, amdS cassette and pRS426 were transformed into yeast for homologous recombination. The resulting plasmid was digested with *Not*I and the amdS marker was replaced with *pyr4* expression cassette, which was liberated from pTTv71 with *Not*I digestion [53]. The *pyr4* expression cassette contained the *pyr4* gene flanked by 310 bp of its upstream region and 225 bp of its downstream region, which was followed by a repeat of the upstream region to facilitate the looping out of the cassette. The deletion plasmid, which was named B7535, was digested with *Pme*I and the fragment containing the integration flanks and *pyr4* marker was gel purified before transformation into *T. reesei*.

*T. reesei* transformants were screened with PCR to confirm the correct integration of the deletion and expression cassettes. 5′ integration was tested with the forward primer targeting the genome region outside of the 5′ flank for the homologous recombination (*pep1* locus: primer PP115, *crt1* locus: C167). The reverse primer targeted the *pdc1* promoter in the case of B6243-based plasmids (PP167) and the *pyr4* promoter (T27) in the case of B7535. For testing 3′ integration, the forward primer targeted the terminator of the *pyr4* gene (T60) and the reverse primer was designed to anneal to the genomic region downstream of the gene and outside of the 3′ flank for the integration (*pep1*: PP115, *crt1*: C168). The absence of the ORF, *pep1* in the case of B6243-based plasmids and *crt1* in the case of B7535, was tested by primers designed to amplify a specific portion of the ORF (*pep1*: PP117 and PP118, *crt1*: C135 and C136). After the initial screening, the positive transformants were plated to trMM + Triton plates for single spore purification. After the purification, the colonies were screened again with the same primers.

### Enzyme and protein assays

Protein concentrations were measured in microtiter plates with Bradford protein reagent concentrate with bovine $\upgamma$-globulin standard (Bio-Rad, Hercules, CA, USA). 10 $\upmu$L of sample and 200 $\upmu$L of the 1:5 diluted reagent were pipetted into wells of 96-well plate and the absorbance at 595 nm was measured 5–30 min after the addition of reagent with Varioskan Flash (Thermo Scientific).

Cellulase activities were measured via assaying 4-methylumbelliferyl-$\upbeta$-d-lactoside (MULac, Carbosynth, Compton, Berkshire, UK) cleavage. Samples were diluted with 50 mM Na-acetate buffer (pH 5.0) and 50 $\upmu$L of the diluted samples were pipetted to the wells of black microtiter plate. Reaction was started by the addition of 50 $\upmu$L 1 mg/mL MULac ($\sim$ 2 mM) and stopped 15 min later by the addition of 100 $\upmu$L 1 M sodium carbonate. Fluorescence was measured with Varioskan Flash (Thermo Scientific) with 355/460 nm excitation and emission wavelengths. The standard curve ranged from 1.25 to 40 $\upmu$M 4-methylumbelliferyl.

Yeast intracellular $\upbeta$-glucosidase activity was measured with methylumbelliferyl-glucoside substrate (Carbosynth). Yeast cultures were harvested in exponential phase and lysed after washing the cells twice with ice-cold water. Cells were resuspended in 100 mM potassium phosphate buffer (pH 6.5 in 20 $^\circ \hbox { C}$) supplemented with protease inhibitor cocktail (cOmplete^TM^ EDTA-free, Roche) before addition of 0.5 mL glass beads (425–600 $\upmu$m, acid-washed) and lysis with Precellys 24 tissue homogenizer (Bertin, Montigny-le-Bretonneux, France) with two 30s cycles at 6500 rpm with 5 min on ice between the cycles. The lysate was clarified by centrifuging 15 min and the activity assayed straight afterwards via the same protocol as described for the MULac assay.

### Sugar uptake assays

Radioactive$^{14}$C-U-glucose (NEC042V250UC) was obtained from PerkinElmer (Waltham, MA, USA) and $^{14}$C-U-lactose (MC 1479) and $^{3}$H-U-cellobiose (MT 1931) from Moravek Biochemicals (Brea, CA, USA). Zero-trans uptake assays were performed as described by Guimaraes et al. [54]. For measuring uptake in yeast, precultures were grown overnight and used to inoculate the main cultures from which yeast cells were harvested in exponential phase by centrifuging with the rotor chilled to 0 $^\circ \hbox { C}$. The cells were washed twice with ice-cold water and resuspended to ice-cold 100 mM potassium phosphate buffer (pH 6.5 in 20 $^\circ \hbox { C}$) to yield a cell suspension with 40–60 OD/mL. Aliquots of the cell suspension and sugar solutions were kept for 5 min in 28 $^\circ \hbox { C}$ water bath before mixing 40 $\upmu$L cells with 20 $\upmu$L 3× sugar solution in a glass tube with conical bottom. After incubation of desired length, the reaction was quenched by pipetting 10 mL of ice-cold water into the tube. The mixture was filtrated with prewetted Whatman GF/C filter paper (GE Healthcare, Helsinki, Finland) and the tube was washed with another 10 mL ice-cold water. The filter paper was transferred into scintillation vial containing 4 mL Ultima Gold XR liquid scintillation cocktail (PerkinElmer) and counted with TriCarb 2810 TR scintillation counter (PerkinElmer). To measure unspecific binding of the sugar to cell surface, 20 $\upmu$L 3× sugar solution and 40 $\upmu$L cells were pipetted into 10 mL ice-cold water, and the resulting solution was filtered and washed immediately afterwards in the same way as normal samples. Linearity was checked by comparing the rate obtained from two different timepoints and from two dilutions of the cell suspension. Uptake rate was calculated by dividing the blank-subtracted counts by reaction time (min), amount of yeast used (based on optical density, for which 1 OD unit equals 0.24 and 0.27 mg$_{\mathrm{CDW}}$/mL for ySS1 and yJR33, respectively) and specific activity of the radioactive stock solution (cpm/nmol). Specific uptake rate was determined as the uptake rate obtained for strains expressing different transporters subtracted with the uptake rate obtained for the negative control strain. Two to three technical replicates were used for each sample.

### RNA extraction and RT-qPCR

Fungal total RNA was extracted with RNeasy plant mini kit (Qiagen, Hilden, Germany), reverse transcribed and the resulting cDNA was analyzed by RT-qPCR. For RNA extraction, sample of mycelium was mixed with 0.5 mL ice-cold glass beads and 0.6 mL RLT buffer supplemented with 1% $\upbeta$-mercaptoethanol, homogenized for 30 s at 6500 rpm with Precellys tissue homogenizer (Bertin instruments, Montigny le Bretonneux, France). RNA was extracted from the supernatant of this extract with the instructions provided with the RNeasy plant mini kit (Qiagen). After extraction and quantification, 0.8–1 $\upmu$g RNA was treated with DNAse I (Thermo-Scientific) before reverse transcription by first strand cDNA synthesis kit (Roche, Basel, Switzerland) with anchored oligo-dT primers. qPCR was done with PowerUP 2× master mix by Thermo Fisher Scientific or with Sybr Green I mix by Roche and *sar1* and *gpd1* were used as a reference genes. The use of *sar1* was suggested by Steiger et al. [55]. Primers were used in 1 $\upmu$M concentration and 2.5 $\upmu$L of the 1:50 diluted cDNA was used as a template in the 20 $\upmu$L reaction. Primer sequences are listed in Additional file 2: Table S2.

The RT-qPCR data was analyzed with the $\Delta \Delta C_\text{p}$ method [56]. In this method, $\Delta C_\text{p}$ is defined as the difference between the C$_{\mathrm{p}}$ values of target and reference genes ($C_{\text{p, target}} - C_{\text{p, ref.}}$). Expression level is defined as $2^{-\Delta C_\text{p}}$, and the fold-change of expression levels between two conditions ($\frac{2^-{\Delta C_{\text{p,inducing}}}}{2^{-\Delta C_{\text{p,non-inducing}}}}$) is termed $\Delta \Delta C_\text{p}$. Log$_{2}$-transformed $\Delta \Delta C_\text{p}$ values were used for comparing the fold-changes between inducing and non-inducing conditions.

### Growth assays

For screening yeast growth on different carbon sources, 6 transformants per strain were picked to wells of an 96-well deep-well plate containing 1 mL SCGE-Ura-His (both glycerol and ethanol at 2%) and grown for 5 days at 28 $^\circ \hbox { C}$ and 800 rpm. 5 $\upmu$L of these precultures were transferred to new plates containing SC-Ura-His medium with different carbon sources. The new plate was incubated for 7 days in the same conditions before the measurement of the final optical density of the cultures.

Bioscreen C microplate incubator (Oy Growth Curves Ab Ltd, Helsinki, Finland) equipped with Bioscreener software was used for characterizing the growth curves of yeast and *T. reesei* strains. Yeast strains were pregrown on SC medium to exponential phase and washed with water before assays. 10–20 $\upmu$L of cell/spore suspension and 200 $\upmu$L of growth medium were added to the wells of the honeycomb plate. The plates were incubated in 28 $^\circ \hbox { C}$ with continuous shaking with normal speed and maximal amplitude. In these conditions, the optical density measured by the Bioscreen machine was discovered to be linear to about OD 6 measured in a conventional spectrophotometer, which corresponded to OD 1.6 in the Bioscreen machine.

### HPLC analysis

HPLC analysis was performed with Waters Alliance Separations Module 2690 (Milford, MA, USA), equipped with Waters 2414 refractive index detector. Cultivation samples were diluted with the eluent (5 mM H$_{2}$SO$_{4}$) and stored in 4 $^\circ \hbox { C}$ before the analysis. Glucose and cellobiose were separated with Fast Acid Analysis and HPX-87H columns (both from Bio-Rad) with 0.5 mL/min flowrate.

## Supplementary information

**Additional file 1.** Additional Figures. Additional figures to the article.

**Additional file 2.** Primer sequences. Sequences of the primers and qPCR primers used in this study.

## Data Availability

The datasets generated during the current study are available from the corresponding author on reasonable request.

## References

[1] Paloheimo M, Haarmann T, Mäkinen S, Vehmaanperä J, Schmoll M, Dattenböck C (2016). Production of industrial enzymes in* Trichoderma reesei*. Gene expression systems in fungi: advancements and applications.

[2] Fonseca LM, Parreiras LS, Murakami MT (2020). Rational engineering of the* Trichoderma reesei* RUT-C30 strain into an industrially relevant platform for cellulase production. Biotechnol Biofuels.

[3] Saloheimo M, Pakula TM (2012). The cargo and the transport system: secreted proteins and protein secretion in* Trichoderma reesei* (Hypocrea jecorina). Microbiology.

[4] Sloothaak J, Tamayo-Ramos JA, Odoni DI, Laothanachareon T, Derntl C, Mach-Aigner AR (2016). Identification and functional characterization of novel xylose transporters from the cell factories Aspergillus niger and* Trichoderma reesei*. Biotechnol Biofuels.

[5] Galagan JE, Calvo SE, Borkovich KA, Selker EU, Read ND, Jaffe D (2003). The genome sequence of the filamentous fungus Neurospora crassa. Nature.

[6] Ramos AS, Chambergo FS, Bonaccorsi ED, Ferreira AJ, Cella N, Gombert AK (2006). Oxygen-and glucose-dependent expression of Trhxt1, a putative glucose transporter gene of* Trichoderma reesei*. Biochemistry.

[7] Shida Y, Furukawa T, Ogasawara W (2016). Deciphering the molecular mechanisms behind cellulase production in* Trichoderma reesei*, the hyper-cellulolytic filamentous fungus. Biosci Biotechnol Biochem.

[8] Galazka JM, Tian C, Beeson WT, Martinez B, Glass NL, Cate JH (2010). Cellodextrin transport in yeast for improved biofuel production. Science.

[9] Znameroski EA, Li X, Tsai JC, Galazka JM, Glass NL, Cate JH (2014). Evidence for transceptor function of cellodextrin transporters in Neurospora crassa. J Biol Chem.

[10] Reis TF, Lima PBA, Parachin NS, Mingossi FB, Oliveira JVC, Ries LNA (2016). Identification and characterization of putative xylose and cellobiose transporters in Aspergillus nidulans. Biotechnol Biofuels.

[11] Kim H, Lee WH, Galazka JM, Cate JH, Jin YS (2014). Analysis of cellodextrin transporters from Neurospora crassa in Saccharomyces cerevisiae for cellobiose fermentation. Appl Microbiol Biotechnol.

[12] Fekete E, Karaffa L, Seiboth B, Fekete É, Kubicek CP, Flipphi M (2012). Identification of a permease gene involved in lactose utilisation in Aspergillus nidulans. Fungal Genet Bbiol.

[13] Zhang W, Kou Y, Xu J, Cao Y, Zhao G, Shao J (2013). Two major facilitator superfamily sugar transporters from* Trichoderma reesei* and their roles in induction of cellulase biosynthesis. J Biol Chem.

[14] Ivanova C, Bååth JA, Seiboth B, Kubicek CP (2013). Systems analysis of lactose metabolism in* Trichoderma reesei* identifies a lactose permease that is essential for cellulase induction. PLoS ONE.

[15] Porciuncula JdO, Furukawa T, Shida Y, Mori K, Kuhara S, Morikawa Y (2013). Identification of major facilitator transporters involved in cellulase production during lactose culture of* Trichoderma reesei* PC-3-7. Biosci Biotechnol Biochem.

[16] Li J, Liu G, Chen M, Li Z, Qin Y, Qu Y (2013). Cellodextrin transporters play important roles in cellulase induction in the cellulolytic fungus Penicillium oxalicum. Appl Microbiol Biotechnol.

[17] Fekete E, Orosz A, Kulcsár L, Kavalecz N, Flipphi M, Karaffa L (2016). Characterization of a second physiologically relevant lactose permease gene (lacpB) in Aspergillus nidulans. Microbiology.

[18] Wang B, Li J, Gao J, Cai P, Han X, Tian C (2017). Identification and characterization of the glucose dual-affinity transport system in Neurospora crassa: pleiotropic roles in nutrient transport, signaling, and carbon catabolite repression. Biotechnol Biofuels.

[19] Martinez D, Berka RM, Henrissat B, Saloheimo M, Arvas M, Baker SE (2008). Genome sequencing and analysis of the biomass-degrading fungus* Trichoderma reesei* (syn. Hypocrea jecorina). Nat Biotechnol.

[20] Jourdier E, Baudry L, Poggi-Parodi D, Vicq Y, Koszul R, Margeot A (2017). Proximity ligation scaffolding and comparison of two* Trichoderma reesei* strains genomes. Biotechnol Biofuels.

[21] Krogh A, Larsson B, Von Heijne G, Sonnhammer EL. Predicting transmembrane protein topology with a hidden Markov model: application to complete genomes. J Mol Biol. 2001;305(3):567–80. www.cbs.dtu.dk/services/TMHMM/. Visited 17 Dec 2019. 10.1006/jmbi.2000.4315 11152613

[22] Beier S, Hinterdobler W, Bazafkan H, Schillinger L, Schmoll M (2020). CLR1 and CLR2 are light dependent regulators of xylanase and pectinase genes in* Trichoderma reesei*. Fungal Genet Biol.

[23] Häkkinen M, Valkonen MJ, Westerholm-Parvinen A, Aro N, Arvas M, Vitikainen M (2014). Screening of candidate regulators for cellulase and hemicellulase production in* Trichoderma reesei* and identification of a factor essential for cellulase production. Biotechnol Biofuels.

[24] Madeira F, Lee J, Buso N, Gur T, Madhusoodanan N, Basutkar P, et al. The EMBL-EBI search and sequence analysis tools APIs in 2019. Nucleic Acids Res. 2019. https://www.ebi.ac.uk/Tools/msa/muscle/. Visited 17 Dec 2019. 10.1093/nar/gkz268 PMC6602479 30976793

[25] Casa-Villegas M, Polaina J, Marín-Navarro J (2018). Cellobiose fermentation by Saccharomyces cerevisiae: comparative analysis of intra versus extracellular sugar hydrolysis. Process Biochem.

[26] Liu JJ, Zhang GC, Oh EJ, Pathanibul P, Turner TL, Jin YS (2016). Lactose fermentation by engineered Saccharomyces cerevisiae capable of fermenting cellobiose. J Biotechnol.

[27] Pakula TM, Salonen K, Uusitalo J, Penttilä M (2005). The effect of specific growth rate on protein synthesis and secretion in the filamentous fungus* Trichoderma reesei*. Microbiology.

[28] Bisson LF, Fan Q, Walker GA, Ramos J, Sychrova H, Kschischo M (2016). Sugar and glycerol transport in Saccharomyces cerevisiae. Yeast membrane transport. Vol. 892 of advances in experimental medicine and biology.

[29] Zhang W, Cao Y, Chen G, Liu W (2017). Identification of the structural determinants for efficient glucose transport via segment swapping between two fungal glucose transporters. RSC Adv.

[30] Sen A, Acosta-Sampson L, Alvaro CG, Ahn JS, Cate JH, Thorner J (2016). Internalization of heterologous sugar transporters by endogenous $$\alpha $$-arrestins in the yeast Saccharomyces cerevisiae. Appl Environ Microbiol.

[31] Peng J, Schwartz D, Elias JE, Thoreen CC, Cheng D, Marsischky G, et al. A proteomics approach to understanding protein ubiquitination. Nat Biotechnol. 2003;21(8):921. www.ubpred.org. Visited 17 Dec 2019. 10.1038/nbt849 12872131

[32] Mikros E, Diallinas G (2019). Tales of tails in transporters. Open Biol.

[33] Stricker AR, Grosstessner-Hain K, Würleitner E, Mach RL (2006). Xyr1 (xylanase regulator 1) regulates both the hydrolytic enzyme system and D-xylose metabolism in Hypocrea jecorina. Eukaryot Cell.

[34] Zhang J, Chen Y, Wu C, Liu P, Wang W, Wei D (2019). The transcription factor ACE3 controls cellulase activities and lactose metabolism via two additional regulators in the fungus* Trichoderma reesei*. J Biol Chem.

[35] Nogueira KM, de Paula RG, Antoniêto ACC, dos Reis TF, Carraro CB, Silva AC (2018). Characterization of a novel sugar transporter involved in sugarcane bagasse degradation in* Trichoderma reesei*. Biotechnol Biofuels.

[36] dos Santos Castro L, de Paula RG, Antoniêto AC, Persinoti GF, Silva-Rocha R, Silva RN (2016). Understanding the role of the master regulator XYR1 in* Trichoderma reesei* by global transcriptional analysis. Front Microbiol.

[37] Antoniêto ACC, dos Santos Castro L, Silva-Rocha R, Persinoti GF, Silva RN (2014). Defining the genome-wide role of CRE1 during carbon catabolite repression in* Trichoderma reesei* using RNA-Seq analysis. Fungal Genet Biol.

[38] Craig JP, Coradetti ST, Starr TL, Glass NL (2015). Direct target network of the Neurospora crassa plant cell wall deconstruction regulators CLR-1, CLR-2, and XLR-1. MBio.

[39] Wu VW, Thieme N, Huberman LB, Dietschmann A, Kowbel DJ, Lee J (2020). The regulatory and transcriptional landscape associated with carbon utilization in a filamentous fungus. Proc Natl Acad Sci.

[40] Kubicek CP, Messner R, Gruber F, Mandels M, Kubicek-Pranz EM (1993). Triggering of cellulase biosynthesis by cellulose in* Trichoderma reesei*. Involvement of a constitutive, sophorose-inducible, glucose-inhibited beta-diglucoside permease. J Biol Chem.

[41] Sternberg D, Mandels GR (1979). Induction of cellulolytic enzymes in* Trichoderma reesei* by sophorose. J Bacteriol.

[42] Fritscher C, Messner R, Kubicek C (1990). Cellobiose metabolism and cellobiohydrolase I biosynthesis by* Trichoderma reesei*. Exp Mycol.

[43] Jourdier E, Cohen C, Poughon L, Larroche C, Monot F, Chaabane FB (2013). Cellulase activity mapping of* Trichoderma reesei* cultivated in sugar mixtures under fed-batch conditions. Biotechnol Biofuels.

[44] Guo B, Sato N, Biely P, Amano Y, Nozaki K (2016). Comparison of catalytic properties of multiple $$\beta $$-glucosidases of* Trichoderma reesei*. Appl Microbiol Biotechnol.

[45] Lee ME, DeLoache WC, Cervantes B, Dueber JE (2015). A highly characterized yeast toolkit for modular, multipart assembly. ACS Synth Biol.

[46] Gietz RD, Woods RA, Xiao W (2006). Yeast transformation by the LiAc/SS carrier DNA/PEG method. Yeast protocol. Methods in molecular biology.

[47] Ma H, Kunes S, Schatz PJ, Botstein D (1987). Plasmid construction by homologous recombination in yeast. Gene.

[48] Winston F, Dollard C, Ricupero-Hovasse SL (1995). Construction of a set of convenient Saccharomyces cerevisiae strains that are isogenic to S288C. Yeast.

[49] Penttilä M, Nevalainen H, Rättö M, Salminen E, Knowles J (1987). A versatile transformation system for the cellulolytic filamentous fungus* Trichoderma reesei*. Gene.

[50] Benson DA, Karsch-Mizrachi I, Lipman DJ, Ostell J, Sayers EW. GenBank. Nucleic acids Res. 2011;39(Database issue):D32. www.ncbi.nlm.nih.gov/genbank/. Visited 28 Jan 2020. 10.1093/nar/gkq1079 PMC3013681 21071399

[51] Sikorski RS, Hieter P (1989). A system of shuttle vectors and yeast host strains designed for efficient manipulation of DNA in Saccharomyces cerevisiae. Genetics.

[52] Landowski C, Huuskonen A, Westerholm-Parvinen A, Saloheimo M, Kanerva A, Hiltunen J. Multiple proteases deficient filamentous fungal cells and methods of use thereof;. US Patent 10,435,731. Filed July 10, 2014 and issued October 8, 2019.

[53] Landowski CP, Huuskonen A, Wahl R, Westerholm-Parvinen A, Kanerva A, Hänninen AL (2015). Enabling low cost biopharmaceuticals: a systematic approach to delete proteases from a well-known protein production host* Trichoderma reesei*. PLoS ONE.

[54] Guimaraes PM, Multanen JP, Domingues L, Teixeira JA, Londesborough J (2008). Stimulation of zero-trans rates of lactose and maltose uptake into yeasts by preincubation with hexose to increase the adenylate energy charge. Appl Environ Microbiol.

[55] Steiger MG, Mach RL, Mach-Aigner AR (2010). An accurate normalization strategy for RT-qPCR in Hypocrea jecorina (*Trichoderma reesei*). J Biotechnol.

[56] Livak KJ, Schmittgen TD (2001). Analysis of relative gene expression data using real-time quantitative PCR and the $$2^{-\Delta \Delta C_T}$$ method. Methods.

