# Supplementary material for: Studies on sugar transporter CRT1 reveal new characteristics that are critical for cellulase induction in Trichoderma reesei
Source: Biotechnol Biofuels. 2020 Sep 14;13:158. doi: 10.1186/s13068-020-01797-7 (PMC7491124; doi:10.1186/s13068-020-01797-7)
Supplement: Supplementary file 1 — Additional file 1. Additional Figures. Additional figures to the article. [file 13068_2020_1797_MOESM1_ESM.pdf]

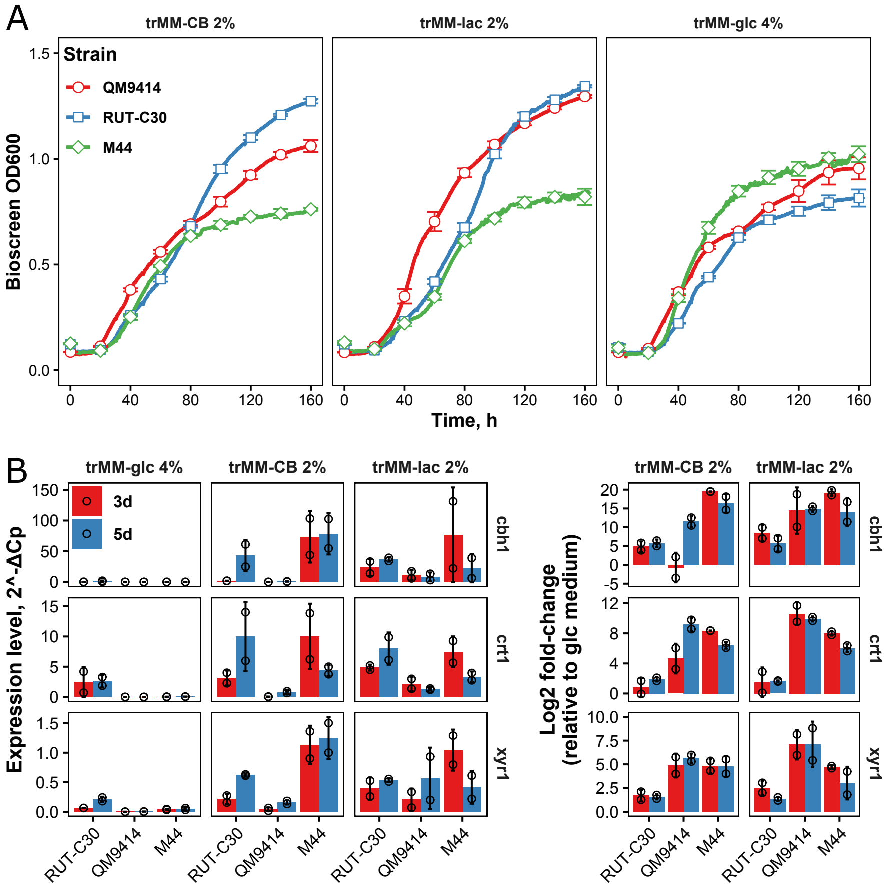

Figure 1 Growth and gene expression analysis of the wild-type strains A Growth curves of the wild-type strains in minimal medium with different carbon sources, measured with the Bioscreen C incubator. Error bars present standard deviation between 3 growth replicates. **B** Expression levels and fold-changes of selected genes from the days 3 and 5 of *T. reesei* MTP cultivation. Error bars present standard deviation between two independent experiments.

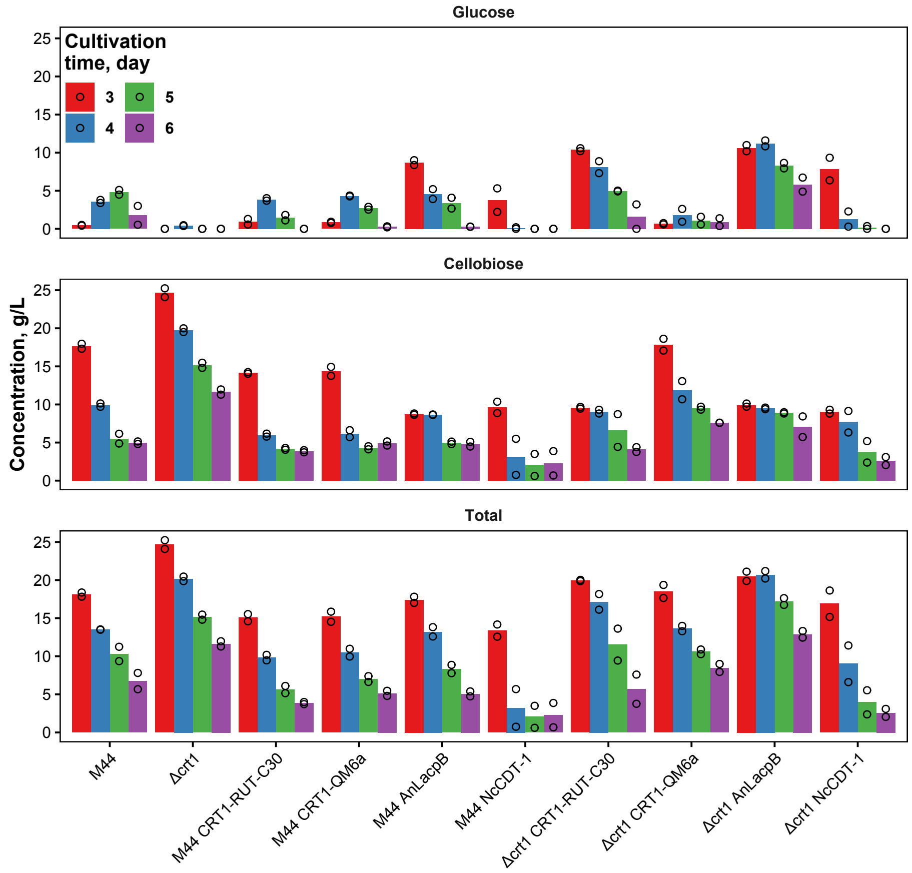

Figure 2 HPLC analysis of culture supernatant from *T. reesei* cultivation Glucose, cellobiose and total sugar (glucose + cellobiose) concentrations from *T. reesei* cultivation on trMM with 2% cellobiose. Measurement points present results of two transformants and bars the average of them.

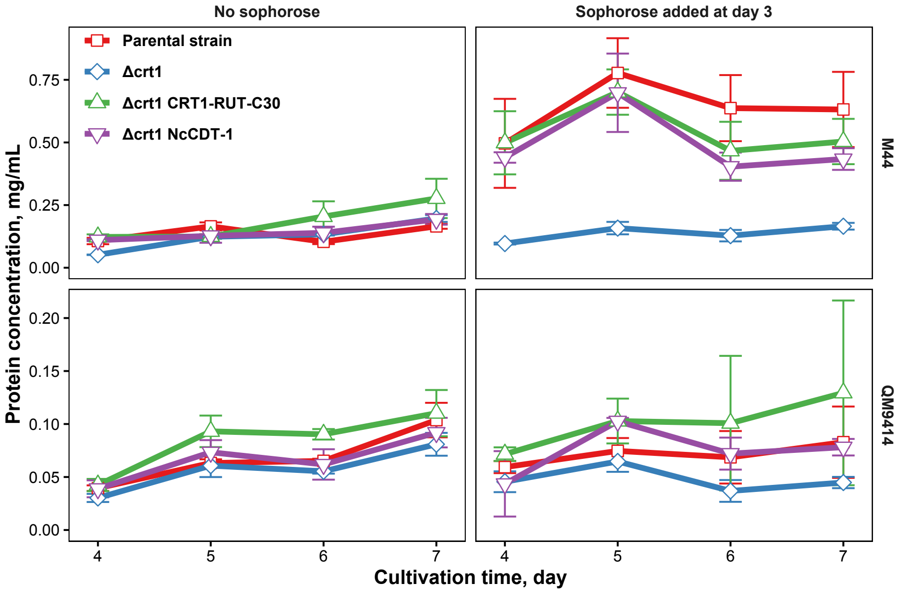

Figure 3 Effect of sophorose addition on protein production by sorbitol-grown strains Protein concentrations from a cultivation where M124 or QM9414-based strains were grown for 3 days on trMM with 2% sorbitol after which sophorose was added to the medium to 0.3 mM final concentration. Error bars present standard deviation between three growth replicates.
