# Supplementary material for: Studies on sugar transporter CRT1 reveal new characteristics that are critical for cellulase induction in Trichoderma reesei
Source: Biotechnol Biofuels. 2020 Sep 14;13:158. doi: 10.1186/s13068-020-01797-7 (PMC7491124; doi:10.1186/s13068-020-01797-7)
Supplement: Supplementary file 2 — Additional file 2. Primer sequences. Sequences of the primers and qPCR primers used in this study. [file 13068_2020_1797_MOESM2_ESM.pdf]

Table 1: Primers used in this article. Bold = flank for recombination, underline = *Pme*I restriction site

Name	Sequence	Description	Usage
SaSS-19	GGTCAATGCAAGAAATACATATTTGGTCT	PGK prom forw	Screening yeast strains for expression plasmid
SaSS-20	CATTCGTGACTATAAAATGAATAAACTAAACTATTC	Eno1 term rev	Screening yeast strains for expression plasmid
SaSS-25	**TTGCCCAGTATTCTTAACCCAACTGCACAGAACAAAAACCTGCAGGAAACGAAGATAAATC**GGAAAGAGTGAGGAACTATCGCATA	PGK prom with Ura3 flank	Integration of the GH1-1 cassette with URA3 flanks
SaSS-27	**TAATTAAATTGAAGCTCTAATTTGTGAGTTTAGTATACATGCATTTACTTATAATACAGTTTTT**ATGATCCGTCGAGTTCAAGAGAAAA	His3 term with Ura3 flank	Integration of the GH1-1 cassette with URA3 flanks
SaSS-30	**AACGATCAAAGCAGCGCAGCTACAGCACAATC**ATGGGCGAGAAAGAAGACATTCACGCTCACG	crt1-rut-c30-pdc-fwd	Expression of RUT-C30 version of crt1 in B6243
SaSS-31	**AACGATCAAAGCAGCGCAGCTACAGCACAATC**ATGATGAAGGAGCCGCCCAAGGCCTGGACC	crt1-qm6a-pdc-fwd	Expression of QM6a version of crt1 in B6243
SaSS-34	**TCTACCGGTGCGTCAGGCTTTCGCCACGGAGCT**TTAAGCCTTCTCGATATTGACAATGTCGCCGTGG	crt1-cbh-term-rev	Expression of crt1 in B6243
SaSS-49	**AAGCACCTCAAAACGATCAAAGCAGCGCAGCTACAGCACAATC**ATGGGTGAGATCAACGAAGAGAAGC	AnLacpB-fwd	AnLacpB expression in B6243
SaSS-50	**CCAAGAATCTACCGGTGCGTCAGGCTTTCGCCACGGAGCT**CTAAGCCTCATCCGCTGAGATCTTCTCGG	AnLacpB-rev	AnLacpB expression in B6243
SaSS-51	**AAGCACCTCAAAACGATCAAAGCAGCGCAGCTACAGCACAATC**ATGTCGTCTCACGGCTCCCATGACG	NcCDT-1-fwd	NcCDT-1 expression in B6243
SaSS-52	**CCAAGAATCTACCGGTGCGTCAGGCTTTCGCCACGGAGCT**CTAAGCAACGATAGCTTCGGACACATGG	NcCDT-1-rev	NcCDT-1 expression in B6243
oSK-186	CCTAGTCCTGTTGCTGCCAA	URA3sc-del-for	Screening yeast strains for GH1-1 integration
oSK-187	ACCGCTAACAATACCTGGGC	URA3sc-del-rev	Screening yeast strains for GH1-1 integration
oSK-192	AGTATTGAGAAGGGCAACGG	URA3sc-5´UTR-for	Screening yeast strains for GH1-1 integration
oSK-193	TTGTTCTTGGAAACGCTGCC	URA3sc-3´UTR-rev	Screening yeast strains for GH1-1 integration
T27	TGCGTCGCCGTCTCGCTCCT	pyr4 5´ rev	Screening *T. reesei* integration
T60	TGACGTACCAGTTGGGATGA	pyr4 3´ fwd	Screening *T. reesei* integration
PP115	CGACGAGAGCCAAGCAGCGC	pep1 int 5´ fwd	Screening *T. reesei* integration
PP116	GCATCCCGTCACCTCCCTAG	pep1 int 3´ rev	Screening *T. reesei* integration
PP117	TTATTTCTGAGCCCAGCCCAGG	pep1 orf 5´	Screening *T. reesei* integration
PP118	GCACCGCCCCCGAGTAACCTCCCACGCCCAC	pep1 orf 3´	Screening *T. reesei* integration
C132	**GTAACGCCAGGGTTTTCCCAGTCACGACG**GTTTAAACAGTCAGCTCGCCTTCTTCAC	crt1 5´ fwd	Construction of the *crt1* deletion plasmid
C133	**CGGTTCTCATCTGGGCTTGCTCGGTCCTGGCGTAGATCTA**GCGGCCGCCACGACCTTGGTCCTGATCT	crt1 5´ rev	Construction of the *crt1* deletion plasmid
C134	**CCGTTTCCAGTGCGGCCGC**GCCACGGACTTTTACTTGCG	crt1 3´ fwd	Construction of the *crt1* deletion plasmid
C135	**GCGGATAACAATTTCACACAGGAAACAGC**GTTTAAACTAAGATACGGATGGCGGCAC	crt1 3´ rev	Construction of the *crt1* deletion plasmid
C136	TAATACGACTCACTATAGCGAGACCAAGGGACGC	crt1 orf 3´ fwd	Screening *T. reesei* integration
C167	TGATGAACTAACCCCAGGTG	crt1 int 5´ fwd	Screening *T. reesei* integration
C168	CATTTCACAGTGCCACGTAAG	crt1 int 3´ rev	Screening *T. reesei* integration

Table 2: qPCR primers used in this article

Gene	Sequence
cbh1	GCGGATCCTCTTTCTCAG
	ATGTTGGCGTAGTAATCATCC
crt1	GTGCTCTCCTTTGAGATGCG
	AAGCTCTTCCAAAGTGCGTC
sar1	TCTCCACCCTACTTCTGAG
	CTTGTTGCCCAGGATGAC
ace3	AGATGATGCAGCACTTTCGAG
	CACTGTATCACGTACTGCTTGG
xyr1	CTGCCGAAGCTATTAGCCAG
	AAAGTTGCGCTGATACTCTGTG
gpd1	TGTCCATTCGTGTCCCTACC
	GTAGGCCAAGATTCCCTTGAG
